# Challenge of Corneal Ulcer Healing: A Novel Conceptual Framework, the “Triad” of Corneal Ulcer Healing/Corneal Neovascularization/Intraocular Pressure, and Avascular Tendon Healing, for Evaluation of Corneal Ulcer Therapy, Therapy of Neovascularization, Glaucoma Therapy, and Pentadecapeptide BPC 157 Efficacy

**DOI:** 10.3390/ph18121822

**Published:** 2025-11-28

**Authors:** Sanja Masnec, Antonio Kokot, Tamara Kralj, Mirna Zlatar, Kristina Loncaric, Marko Sablic, Miro Kalauz, Iva Beslic, Katarina Oroz, Bozana Mrvelj, Lidija Beketic Oreskovic, Ivana Oreskovic, Sanja Strbe, Borna Staresinic, Goran Slivsek, Alenka Boban Blagaic, Sven Seiwerth, Anita Skrtic, Predrag Sikiric

**Affiliations:** 1 Department of Pharmacology, School of Medicine, University of Zagreb, 10000 Zagreb, Croatia; sanjamp@yahoo.com (S.M.); tamara_kralj@yahoo.com (T.K.); goran.slivsek@xnet.hr (G.S.); abblagaic@mef.hr (A.B.B.); 2 Department of Ophthalmology, University Hospital Center Zagreb, School of Medicine, University of Zagreb, 10000 Zagreb, Croatia; 3 Department of Anatomy and Neuroscience, School of Medicine Osijek, University of Osijek, 31000 Osijek, Croatia; msablic@mefos.hr; 4 Department of Pathology, School of Medicine, University of Zagreb, 10000 Zagreb, Croatia

**Keywords:** corneal ulcer healing, corneal neovascularization, intraocular pressure, tendon healing, evaluation, corneal ulcer therapy, therapy of neovascularization, glaucoma therapy, pentadecapeptide BPC 157

## Abstract

To better address the challenge of corneal ulcer healing, with already available standard agents, and those recently introduced, such as stable gastric pentadecapeptide BPC 157, we introduced a novel conceptual framework—the “triad” of corneal ulcer healing↔corneal neovascularization↔intraocular pressure—and extended it to avascular tissues such as tendon. Within this framework, cytoprotection serves as the unifying principle, underscoring that therapeutic effects are not isolated but interconnected. Preclinical studies with BPC 157 therapy, as a cytoprotection agent, illustrate this integration. BPC 157 rapidly normalizes elevated intraocular pressure in glaucomatous rats, preserves retinal integrity, restores pupil function, maintains corneal transparency during ulcer or abrasion healing, and counteracts both corneal neovascularization and dry eye. In parallel, its consistent efficacy in tendon injury models highlights a cytoprotective specificity across avascular tissues. The cornea’s “angiogenic privilege,” preserved during healing and tendon recovery together, provides strong proof of concept. Furthermore, mapping standard therapeutic agents used for corneal ulcers, neovascularization, or glaucoma onto this triad, and linking them with tendon healing, reveals both shared pathways and inconsistencies across existing drug classes. Analyzed were the ascorbate, fibronectin, hyaluronic acid, metalloproteinase inhibitors, EGF, FGF, NGF, insulin, and IGF-1 (corneal ulcer healing), the antiangiogenic agents (endostatin, PAI-1, PEDF, angiostatin, TSP-1, TSP-2, IFN-α), corticosteroids, NSAIDs, cyclosporine A, anti-VEGF drops (treatment of corneal neovascularization), and alpha 2-agonists, beta-blockers, carboanhydrase inhibitors, muscarinic agonists, Rho-kinase inhibitors, and prostaglandin analogs (glaucoma). Taken together, these findings advance cytoprotection as a unifying therapeutic paradigm, with BPC 157 emerging as its first exemplar, and encourage further translational research toward clinical application.

## 1. Introduction

Better resolution of corneal ulcer healing with pharmacotherapy is a pertinent challenge. To this point, this review attempts to promote a new therapy way, based on the available standard agents [1,2], and those recently introduced (i.e., stable gastric pentadecapeptide BPC 157 [3]). This would be sharing particular interconnections, an essential “triad”, corneal ulcer/wound healing, corneal neovascularization, and intraocular pressure.

Recently, as a cytoprotective agent therapy [4,5,6,7,8], the stable gastric pentadecapeptide BPC 157 was reviewed in glaucoma [9], and in other tissues [10,11]. This was providing pleiotropic beneficial effects [12], a long-known conceptual characteristic of cytoprotective agents as pointed out in funding cytoprotection papers (i.e., [13,14,15,16,17,18,19,20,21]), and, especially, in the particulars of wound healing [22,23,24,25]. With this new conceptual perspective, for corneal ulcer healing, this therapy perspective was now reviewed compared to standard existing eye therapies (i.e., anti-VEGF agents, corticosteroids, EGF, NGF, and others) used in corneal ulcer healing, therapy of neovascularization, and glaucoma therapy. This was carried out using the mentioned novel “triad” approach (corneal ulcer healing↔corneal neovascularization↔intraocular pressure). Notably, this may be important, providing that the standard agents act in “fragments” and conceptually emphasize why these fragmented approaches fail to prevent cross-effects (e.g., anti-VEGF may impair ulcer healing [26], corticosteroids may worsen intraocular pressure [27,28], etc.).

To highlight the eye therapy in general, and corneal ulcer therapy, in particular, it may be a unified framework (“cytoprotection”) that conceptually links corneal ulcer healing, corneal neovascularization, and intraocular pressure regulation. This is a fresh perspective compared to the traditional fragmented approaches, where these phenomena are usually studied and treated in isolation [1,2]. Positioning them as a “triad” is a compelling way to highlight their interdependence. Likewise, this “triad” could hallmark the full efficacy and potential of the agent used in either eye therapy. Highly regarded in the literature, but never practically implemented, there is the concept of cytoprotection, as one of the most precise holistic concepts (i.e., innate cell protection against diverse noxious events toward original status, and therefore, integrated beneficial agents’ effects [13,14,15,16,17,18,19,20,21]).

**Figure 1:**
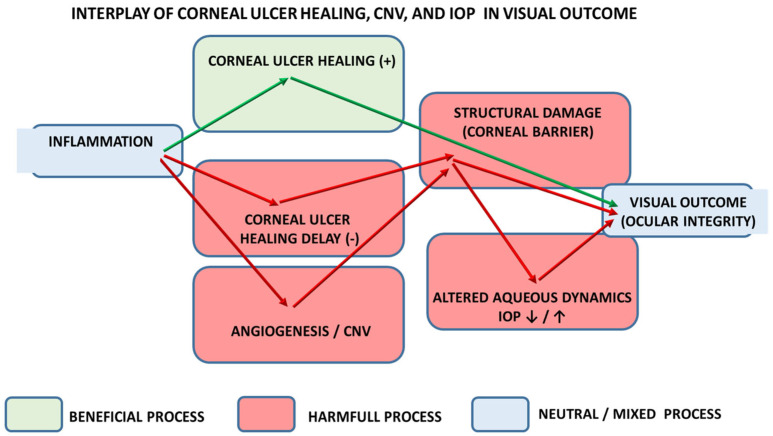
Cytoprotective triad: corneal ulcer healing, corneal neovascularization (CNV) control, and intraocular pressure (IOP) regulation. Tendon healing (avascular parallel) is included as an external validation axis for avascular tissue-specific efficacy.

While focusing on corneal ulcer healing, this review, based on the different agents’ effects and use in eye therapy (corneal ulcer, or corneal neovascularization, or increased intraocular pressure), considers the general evidence that corneal ulcer healing, corneal neovascularization, and intraocular pressure are interrelated [1,2,29,30,31,32,33,34], a “triad”, and together they strongly influence ocular integrity and visual outcome. As commonly acknowledged, they act through linked biological and mechanical pathways (inflammation→angiogenesis→altered aqueous dynamics/structural damage), so problems in one domain commonly affect the others. Thereby, not only in theory, but more in practice, for long-term ocular integrity (i.e., clear, structurally stable cornea) and optimal visual outcome (i.e., preservation of transparency and optic nerve function), conceptually, the corneal ulcer healing (e.g., ↑)↔corneal neovascularization (e.g., ↓)↔intraocular pressure (e.g., ↓) relation is integrated in one agent’s effect. At best, such balanced therapy simultaneously promotes epithelial/stromal repair, suppresses pathological neovascularization, and maintains or recovers physiological intraocular pressure. In a broader context, the particular aspect of that system (corneal transparency/avascularity), the potential of drugs and drug classes supposed to activate or inhibit the system by interacting depending on circumstances, with specific molecular targets, highlights the corresponding effect on other tissue healing, particularly those avascular (i.e., tendon) [4] as an “external validation axis”. Thereby, evidenced tendon healing should be regarded as a hallmark of the well-orchestrated effect [4] (see Figure 1).

Notably, this tendon/cornea avascular analogy as a clue could serve as an independent validation axis for agents that modulate angiogenesis and matrix remodeling [4]. Certainly, it departs from current therapy-specific terms of either ulcer healing, or the therapy of neovascularization, or the decrease in intraocular pressure [1,2,29,30,31,32,33,34], since the current eye therapy does not consider musculoskeletal disorders [1,2,29,30,31,32,33,34]. As a crucial advantage [4], defining the agent’s activity also by tendon healing (avascular analogy) may reveal a particular aspect of corneal ulcer healing itself. Furthermore, such additional (in)direct characterization of corneal ulcer healing [4] would exemplify positive and negative innate interconnections between corneal ulcer/neovascularization/intraocular pressure, revealing particularities of the “triad” as a print of the agent’s activity.

In this context, recently, we have attempted to implement the cytoprotection concept in eye therapy [9]. The evidence, still preclinical, was reviewed as a novel insight that the stable gastric pentadecapeptide BPC 157 therapy can recover glaucomatous rats [9], rapidly lower increased intraocular pressure to normalize intraocular pressure, maintain retinal integrity [35], and recover pupil function [36]. Accordingly, it recovers retinal ischemia [37]. Likewise, it heals corneal injuries (i.e., maintains transparency after complete corneal abrasion [38] and corneal ulceration [3], and counteracts dry eye after lacrimal gland removal [39] or corneal insensitivity [40]) (Table 1).

Practically, for eye therapy, particularly in the context of corneal ulcer healing, this conceptual cytoprotective approach can be beneficial. Notably, this may be for stomach ulcer~corneal ulcer [3]. Since 1979, the concept of cytoprotection has evolved from a breakthrough management strategy for stomach ulcers to a broader therapeutic approach, encompassing organoprotection (i.e., cytoprotection→organoprotection). It holds as a novel therapy, the maintaining and reestablishing original gastric integrity via the orchestrated protection of epithelial and endothelial cells. In practice, via cytoprotection agent application, the cytoprotection concept is proven and translated to other organ therapies (cytoprotection→organoprotection) [13,14,15,16,17,18,19,20,21]. An indicative analogy is prostaglandin analogs, i.e., latanoprost, key glaucoma therapy [41], as prostaglandins were the first mediators of cytoprotection as a concept of general (healing) significance, directly preventing epithelial necrosis that may arise in the stomach from the direct injurious effect of various agents’ applications, and thereby in other tissues as well [13,14,15,16,17,18,19,20,21]. Therefore, conceptually, there is a pleiotropic beneficial effect for cytoprotection agents [13,14,15,16,17,18,19,20,21], and for BPC 157, easily applicable, as a supposed cytoprotection mediator, as native and stable in human gastric juice, in particular [4,5,6,7,8,9,10,11,12,23,24,25,26]. Notably, recently, BPC 157 pleiotropic effects were reviewed as a therapy and safety key [4]. A special beneficial effect occurs by controlling and modulating angiogenesis and NO-system, targeting angiogenesis and NO’s cytotoxic and damaging actions, but maintaining, promoting, or recovering their essential protective functions, thus, cornea healing “angiogenic privilege” along with advanced tendon healing as a proof of the concept [4].

This cytoprotection translational evidence [4], taken together, should be crucial, especially in the context of corneal ulcer healing (corneal avascularity vs. corneal neovascularization; corneal healing vs. healing in other organs). From theory to practice, these cytoprotection terms should include all points mentioned, as noted with BPC 157 eye therapy. These were glaucomatous rats’ recovery [9,35], increased intraocular pressure promptly lowered and normalized [35], and the recovery of retinal integrity [35,37], pupil function [35,36], and retinal ischemia [37]. BPC 157 therapy maintained transparency while healing of corneal injuries [3,38,39,40], and counteraction of dry eye and corneal insensitivity occurred while maintaining tear production [39,40]. Linking with tendon healing assures the needed specificity of the therapy [4] (note, BPC 157 therapy’s beneficial effects are confirmed in several tendon injury models [42,43,44,45,46,47,48,49]).

These should be viewed in the context of a persistent attempt to develop more effective corneal management, particularly for corneal ulcers, given the numerous reviews that reanalyze all the particularities of corneal wound healing as a complex process, although, without combining with cytoprotection theory. Notably, this was without referring to the integration of corneal ulcer healing/corneal neovascularization/intraocular pressure for therapy’s purpose, or tendon healing as a hallmark of an orchestrated healing capacity. The general focus was on the significance of the corneal anatomy, the mechanisms of corneal epithelial and stromal wound healing, and endothelial dysfunction (i.e., one of the most common causes of corneal blindness) [50]. Then, the focus was on decreased corneal sensation that may indicate an underlying neurotrophic ulcer or herpetic ulcer [51,52], coordinated interaction of corneal cellular elements, a variety of cytoactive factors, components of the extracellular matrix (ECM), and biomechanical forces such as eyelid movement, and soluble factors known to play major roles in modulating these processes [1,33,53,54,55,56]. Likewise, with no integrating attempts, the other focus was on opposing neovascularization and the therapy [1,56,57,58,59]. In the same way, the additional sole focus was on the elevated intraocular pressure, which may be commonly present [29,30,60,61].

Therefore, corneal ulcer healing/corneal neovascularization/intraocular pressure, the “triad”, remains to be further redefined given the full effectiveness implications for the agents implicated in corneal healing, and corneal ulcer, in particular.

Moreover, as we recently elaborated [4], the credibility of these cytoprotection requirements is challenged by a common requirement for adverse effects. Likewise, there is widespread understanding that any of the therapeutic interventions have side effects. Contrarily, the concept holds that there is such general therapy involving all organs, together and in particular, that should not produce any harm on the other side. Cytoprotection is conceived as a mechanism that, in principle, should avoid collateral injury because it restores homeostasis rather than overriding physiology [13,14]. Conceptually, given that a cytoprotection agent, by definition, should not produce any adverse effect on account of its beneficial effects (although this is not the case with prostaglandins), the corneal ulcer healing/corneal neovascularization/intraocular pressure controlling relations should also link the avascular tendon. Thereby, it would depict the healing depending on timing and tissue specificity [4]. Thus, augmenting ocular cytoprotection with tendon-healing readouts provides an avascular tissue benchmark that clarifies an agent’s capacity to restore homeostasis in corneal ulcer disease, enabling more mechanism-driven translation [4].

Likewise, this implicates high effectiveness (i.e., BPC 157 therapy is effective in the 10 µg–10 ng/kg range). In toxicology studies, BPC 157 exhibited a negative limit test, 2 g/kg i.v. or i.g., without adverse effects in mice, and a lethal dose (LD1) was not achieved [4,5,6,7,8,9,10,11,12,23,24,25,26]. Later, it was effectively used in ulcerative colitis trials (phase II) without adverse effects [62,63], and quite recently, in knee pain [64] and interstitial cystitis [65]. Thus, together, these clinical human data can join the large range of beneficial effects of the BPC 157 therapy indicated by animal experiments, although the human studies performed so far are scarce, with limitations in some of them (i.e., they lacked a large sample size, ethnic variation, and a sham control group). However, these clinical studies effectively encompassed a wide range of investigations (for additional points in musculoskeletal disorders, see [66,67]). Consistently, there are similar beneficial results of BPC 157 therapy obtained after different application routes. Thus, such high applicability of the agent [4,5,6,7,8,9,10,11,12,23,24,25,26] (i.e., native and stable in human gastric juice for more than 24 h [68]), overwhelms the standard cytoprotective agents’ limited (prophylactic application only) applicability and activity [4,5,6,7,8,9,10,11,12,23,24,25,26]. This clearly shows the avoidance of pitfalls already indicated during the concept’s establishment [13,14,15,16,17,18,19,20,21], and it attempts [4,5,6,7,8,9,10,11,12,23,24,25,26] to realize the concept’s tools.

All of these points may be supported by its special interaction with various molecular pathways [69,70,71,72,73,74,75,76,77,78,79,80]. This includes, as a major point, the NO-system [70,71,72] as a whole, counteraction of the adverse effect of either NO-blockade or NO-overactivity, demonstrated in more than 80 targets (i.e., hypertension, hypotension, thrombosis, bleeding) investigated [81,82,83,84,85].

At least partly, this explains the cytoprotective ability of the pentadecapeptide BPC 157 to further maintain and upgrade endothelium integrity and functioning (for review see [4]), particularly focused on minor vessels during noxious procedures, rapid upgrading, and collateral pathways’ activation to substitute the function of the failed major blood vessels [4]. Illustratively, this likely occurred in glaucomatous rats, where one episcleral vein remained and took over the failed function of all the episcleral veins, amid rapid lowering of increased intraocular pressure as proof [9,35].

## 2. Corneal Ulcer Healing Therapy in Terms of “Triad” Approach (Corneal Ulcer Healing↔Corneal Neovascularization↔Intraocular Pressure)

Beyond the well-documented evidence of diverse etiologies of corneal ulceration, current therapy is largely etiology-specific. For infectious ulcers, antibiotics are used against bacteria, antivirals against viruses (predominantly herpes simplex), antifungal drugs for fungal ulcers, and combined antiseptic/anti-amoebozoal therapy for parasitic infections such as Acanthamoeba. For non-infectious causes, treatment is directed at the underlying condition, such as lubricating drops for dry eye or immunosuppressants for autoimmune disorders [86]. In parallel, numerous trials have aimed to enhance corneal wound healing through topically applied agents, most commonly ascorbate, fibronectin, hyaluronic acid, metalloproteinase (MMP) inhibitors, EGF, fibroblast growth factor (FGF), NGF, insulin, and insulin-like growth factor-1 (IGF-1). However, as already emphasized, despite these efforts, no analysis has yet addressed the integrated relationship between corneal ulcer healing, corneal neovascularization, and intraocular pressure, nor has this been linked to healing in other avascular tissues (e.g., tendon), which could serve as a valuable comparative model and a healing highlight.

### 2.1. Ascorbate

A considerable number of manuscripts showed corneal benefit from topical/systemic ascorbate and corneal ulcer healing. Ascorbate speeds epithelial re-closure, and lowers inflammatory cell influx and neovascularization in many models. Thus, (healing + reduced neovascularization) is supported in multiple in vivo corneal models (alkali and thermal burns, epithelial debridement, suture/cauterization models); topical ascorbate (commonly 10% or other concentrations depending on the model) accelerates epithelial repair or reduces stromal ulceration and—importantly—reduces corneal neovascularization in experimental models [87,88,89,90,91,92,93,94,95,96].

Furthermore, considering (healing + reduced neovascularization + reduced intraocular pressure), the published experimental and clinical literature suggests either no effect or, more often, an intraocular pressure-lowering/intraocular pressure-modulating effect of ascorbate (including IV or very high oral doses producing transient osmotic intraocular pressure drops) [84,88,89,90,91,92,93,94,95,96].

Finally, considering the final extension (healing + reduced neovascularization + reduced intraocular pressure + tendon healing), ascorbic acid/vitamin C accelerates tendon healing associated with early angiogenesis or changes in angiogenic markers [97,98,99,100,101,102,103]. The preclinical evidence is consistent in rat, rabbit, and chicken models + in-vitro tenocyte/tendon-derived stem cells work, as reported acceleration of tendon repair besides earlier angiogenesis includes increased fibroblast/tenocyte activity, greater type I collagen production, larger collagen fibril diameter, better collagen organization, and improved biomechanical strength.

### 2.2. Fibronectin

Fibronectin is a large extracellular matrix glycoprotein with a molecular weight of approximately 500–600 kDa. It interacts with integrins—transmembrane receptor proteins—as well as other matrix components, including collagen, fibrin, and heparan sulfate proteoglycans such as syndecans. Fibronectin healing of corneal ulcer in rabbits and patients (purified from autologous plasma by affinity chromatography and administered topically) [104,105,106,107,108,109,110,111,112,113,114,115,116,117] was ascribed to facilitation of corneal epithelial migration. Aiding surface re-epithelialization and decreasing corneal ulceration was ascribed to a prominent fibronectin–fibrinogen matrix, which remained on the surface of fibronectin-treated corneas. There is no direct evidence demonstrating that topically applied full-length plasma fibronectin induces de novo vessel formation in an otherwise normal cornea. However, considering corneal neovascularization, fibronectin is recognized as a pro-angiogenic molecule. Multiple lines of evidence support a causal role of fibronectin in ocular angiogenesis, implicating fibronectin itself, its domains or fragments, and its receptors in the regulation of angiogenic processes within the cornea and other ocular tissues [118,119,120].

There, fibronectin-1 is consistently elevated in vascularized corneas, implicating a pathologic neovascularization process, particularly via endothelial and immune cell interactions [121]. Moreover, considering fibronectin (corneal healing (↑) + corneal neovascularization (↑)) to intraocular pressure, many studies (in)directly link fibronectin/fibronectin fibrils to increased intraocular pressure or trabecular meshwork dysfunction. The increased density of fibronectin fibrils is commonly thought to increase intraocular pressure by altering the compliance of the trabecular meshwork [122,123,124,125,126,127,128]. In rats, fibronectin alone can chronically elevate intraocular pressure and induce neurodegenerative changes given a single intracameral injection of biodegradable poly(lactic-co-glycolic acid (PLGA) microspheres loaded with fibronectin [129].

Finally, fibronectin/tendon healing/angiogenesis is consistent with a lack of direct evidence for fibronectin (corneal healing (↑) + corneal neovascularization (↑) + intraocular pressure (↑)), since there is no evidence that direct application of fibronectin alone (i.e., exogenous fibronectin as the sole therapeutic agent, without concurrent growth factors, cells, scaffolds or gene delivery) is sufficient to produce functional tendon healing and angiogenesis in vivo.

However, toward improved tendon healing is the evidence that bFGF/PDGF and other biologics increase fibronectin deposition in the provisional matrix and promote revascularization/angiogenesis during tendon healing [130].

### 2.3. Sodium Hyaluronan, Hyaluronic Acid

Hyaluronic acid is a naturally occurring glycosaminoglycan that, by virtue of its viscosity, elasticity, and other rheological properties (particularly for its biocompatibility), acts as a lubricating and shock-absorbing fluid in skin and joints and as an ocular lubricant [131]. The supportive background goes with hyaluronic acid as a major component of the extracellular matrix, which is supposed to play a key role in tissue regeneration, inflammation response, and angiogenesis, which are phases of wound repair [132]. Consistently, hyaluronic acid successfully accelerates re-epithelialization of corneal wounds in both animal and human studies [133,134,135,136,137,138,139,140,141,142].

As an additional important point, hyaluronic acid did not induce corneal neovascularization in rabbits [143], and in rat models of alkali-induced corneal burns, cross-linked and standard linear hyaluronic acid hydrogels, delivered via in situ forming hydrogels, resulted in faster re-epithelialization, lower stromal inflammation, and significantly reduced neovascularization [144].

On the other hand, the viscoelastic properties of hyaluronic acid on liquid connective tissue have been proposed for the treatment of tendinopathies, and tendon healing combined with the improved angiogenesis [145,146,147,148,149].

Contrarily, thought to be a large molecule, hyaluronic acid may impair trabecular meshwork outflow by clogging channels, thereby reducing aqueous humor drainage. Hyaluronic acid increased intraocular pressure in both animal and clinical settings [150,151,152]. Illustratively, acute intracameral injection of 1% hyaluronic acid induced a twofold increase in intraocular pressure in rats [150].

### 2.4. MMP Inhibitors

The significance of MMP inhibitors is tied to the general importance of the MMP family, which consists of enzymes involved in the degradation of the extracellular matrix in tissues. In the skin, keratinocytes and fibroblasts express various MMPs during healing [152]. Similarly, corneal epithelial cells and keratocytes secrete MMPs during healing following various corneal injuries. Overexpression in infectious keratitis (bacterial, fungal, viral, Acanthamoeba) occurs with driving collagen degradation, stromal melting, thinning, and potential perforation [153]. Moreover, there is interleukin (IL)-1β—amplified MMP production by corneal fibroblasts and tissue destruction even after pathogens are eradicated [154].

Thus, as an MMP inhibitor, doxocycline is illustrative. At sub-antimicrobial doses, doxycycline inhibits collagenases and MMPs in vitro. Systemic administration in rabbit models significantly reduced corneal ulcer incidence—from 85% to 9% after alkali burn ulceration—and halved perforation rates in Pseudomonas ulcers [155]. Likewise, a similar beneficial effect on corneal ulcer healing occurred with other MMP inhibitors [156,157,158]. Additional experimental agents include ascorbic acid, epigallocatechin gallate (EGCG) (green tea polyphenol), urokinase plasminogen activator (uPA)-plasmin inhibitors, corticosteroids, and even tumor necrosis factor (TNF)-α blockers like infliximab, all investigated for indirect or direct MMP-suppressive or anti-collagenolytic effects [154]. A particular point occurs with galardin, as a hydroxamate broad-spectrum MMP inhibitor with potential in preclinical settings—since concerns considering MMP-system general activity include impaired normal wound healing, tumor-related effects, and other off-target impacts [153].

MMP activity promotes corneal angiogenesis. Thereby, MMP inhibitors, in principle, inhibit corneal angiogenesis [158]. However, it may be an opposite potential theoretical point for metalloproteinase inhibitors. MMP-12, a macrophage-associated protease, inhibits corneal inflammation and neovascularization by regulating CCL2/CCR2 chemokine pathways, based on murine injury models [159,160]. Thus, inhibition of MMP-12 might theoretically worsen inflammation and neovascularization—though such induced corneal neovascularization via inhibition has not been directly tested [161].

On the other hand, systemic doxycycline (a broad-spectrum gelatinase inhibitor) weakened rat Achilles tendons post-injury, indicating that inhibiting necessary MMP activity early may impair biomechanical healing [162].

Commonly, MMPs support intraocular pressure homeostasis in the trabecular meshwork. In the trabecular meshwork (TM) of the eye, endogenous MMPs (e.g., MMP-1, MMP-2, MMP-3, MMP-14) regulate ECM turnover, thereby maintaining aqueous humor outflow and stabilizing intraocular pressure [163]. An imbalance favoring MMP inhibition (e.g., excessive TIMP activity) contributes to ECM accumulation, increased outflow resistance, and elevated intraocular pressure in models of glaucoma and ocular hypertension [163,164,165].

### 2.5. EGF

Since the very beginning, EGF has shared general significance with other growth factors, such as bFGF, PDGF, and VEGF, which are endogenously derived and are thought to play a role in the natural history of healing, having considerable therapeutic potency. Illustrating their presentation (i.e., in tear fluid [166], corneal epithelium [167]) and consequent potential activity as a general point, an instructive emphasis was made in gastrointestinal research; these relatively large peptides are active in ng quantities, and their molar potency is 2–7 million times superior to cimetidine-like drugs [168,169,170]. This might be related to the fact that these growth factors, i.e., in duodenal ulcer healing, stimulate with varying potency virtually all the cellular elements needed for ulcer healing, e.g., epithelial cell proliferation and migration by EGF > bFGF > PDGF, fibroblast proliferation by bFGF > PDGF, and angiogenesis by VEGF > bFGF >> PDGF >> EGF [168,169,170]. Thereby, in particular for corneal ulcer healing, in analogy with skin healing [171], activation of EGFR in corneal cells has been shown to be crucial for the regeneration of the epithelium, stroma, and endothelium after corneal injury. However, the molecular mechanisms involved remain largely unclear beyond what is known in skin tissue [167]. EGF promotes the synthesis of specific proteins and supports the proliferation and differentiation of corneal epithelial cells, keratocytes, and endothelial cells. Moreover, the EGF family of growth factors plays a key role in stimulating lacrimal gland secretion. EGF and EGF-like proteins also interact with neurotransmitter-activated pathways, enhancing both the volume and composition of tear production [172,173]. Thus, since the very beginning, there has been a considerable number of reports on EGF on corneal ulcer healing in animals [171,174,175,176,177,178,179,180] and humans [181,182,183,184,185].

Contrarily, EGF enhances and induces corneal neovascularization [186,187,188].

There is no evidence that exogenous EGF application affected intraocular pressure or caused an increase in intraocular pressure [189]. EGFR expression increases in response to intraocular pressure elevation upregulation in astrocytes, linked with NOS-2 and MMP expression, potentially contributing to neurodegeneration [190].

Finally, considering the skin/cornea analogy, the evidence that exogenous administration of recombinant human epidermal growth factor (rhEGF; also called nepidermin) accelerates wound re-epithelialization, angiogenesis, fibroblast proliferation, and collagen deposition is confronted with the fact that free EGF is unstable. Thus, as rapidly degraded by proteases, and cleared, therefore, intelligent delivery carriers are essential for therapeutic efficacy [191,192,193,194]. However, exogenous EGF (human recombinant EGF or hrEGF) (intralesional application) was applied and reported improved tendon/tendon-repair histology with increased vascularity [195,196], providing histological and immunohistochemical (vascularity, cellularity, collagen organization), while biomechanical improvements were mixed/absent (histology improved but not always tensile strength).

### 2.6. FGF

For FGF, the corneal ulcer healing–corneal neovascularization–intraocular pressure relation, and finally, tendon healing as a hallmark, should be regarded with complex evidence. FGF has been given essential importance as a family of cell signaling proteins, as they are implicated in mediating various processes, such as angiogenesis, wound healing, metabolic regulation, and embryonic development, through their specific receptors. FGF can stimulate angiogenesis and proliferation of fibroblasts, and it is a powerful angiogenesis factor. Twenty-three subtypes have been identified and divided into seven subfamilies. Therefore, there is a possible application in wound healing with good therapeutic effects, i.e., topically to diabetic foot ulcer, with a particular focus on FGF-1, FGF-2, FGF-4, FGF-7, FGF-21, and FGF-23 [197].

Likewise, along with reduced inflammatory cytokines (IL-6, TNF-α, MMP-2/9), increased anti-inflammatory IL-10 and antioxidant SOD-1, and improved tear production and nerve regeneration, corneal ulcer healing is consistently established with topical FGF application in several models, in animal and human studies, including topical recombinant human FGF-21, FGF-7, FGF-2, and recombinant human bFGF [198,199,200,201]. Illustratively, treatment with bFGF alone accelerated epithelial healing, and the combination with CCP (an antioxidant enzyme) further enhanced closure rates and reduced oxidative damage [202]. A comparative evaluation in rabbits that assessed recombinant human EGF versus basic FGF on corneal epithelial wound healing found that bFGF promoted epithelial cell proliferation and migration, similar to EGF, with enhanced healing dynamics [203].

Contrarily, along with increased VEGF-A, C, and D expression, amplifying angiogenic signaling cascades, FGF-2 (bFGF) is one of the most potent angiogenic factors in the cornea [56]. Following corneal injury, it is significantly upregulated and directly stimulates corneal vascular endothelial cells, promoting both blood and lymphatic vessel formation [204]. Illustratively, in classic corneal micropocket assays using pellets, FGF-2 alone reliably induces both angiogenesis and lymphangiogenesis in a dose-dependent manner [205] as well as in corneal suture and HSV keratitis [206,207].

Finally, considering FGF’s influence on intraocular pressure, in young chicks, intraocular injections of IGF-1 plus FGF-2 induced dose-dependent increases in intraocular pressure, along with ocular enlargement, anterior chamber shallowing, and lens thickening [208].

On the other hand, stimulation of angiogenesis is commonly thought to be essential for FGF therapy in tendon injuries, where the increase in both cellularity and vascularization corresponded with earlier functional recovery [209,210,211,212,213,214,215]. However, the effectiveness and delivery strategies are based on the scaffold-based release systems or cell/gene vectors [216].

### 2.7. NGF

At the general level, there is considerable significance of NGF, as a soluble protein produced by and acting upon many different cells located in the nervous, endocrine, and immune systems. It stems from exerting a critical role on epithelial cells and fibroblasts under normal and pathological conditions. NGF is constitutively expressed in human and rat corneas; all major corneal cells—including epithelial, stromal, endothelial, and limbal stem cells [217,218,219]. Consistently, along with promoting epithelial cell proliferation/migration, enhancing nerve survival and regeneration, regulating inflammation, facilitating fibroblast activity and angiogenesis, and restoring tear function, topical NGF significantly accelerated epithelial and stromal healing in animal models and human organ culture, restoring corneal integrity through induction of receptor-mediated signaling [219]. As a particular point, NGF enhances the expression of MMP-9 and cell migration in the epithelium, which are also key to corneal neovascularization pathogenesis [217].

However, to date, induction of corneal neovascularization has not been reported in clinical and preclinical studies specific to NGF in the cornea [220,221,222,223]. Indicatively, in human patients treated with topical NGF (murine or rhNGF), healing occurred without evidence of neovascularization—even in inflammatory and melting ulcers otherwise prone to vascular ingrowth [224].

There is no study demonstrating that applying NGF lowers an already-elevated intraocular pressure. Studies applying NGF to the eye (i.e., topical recombinant human NGF (rhNGF) or through conjunctival or retrobulbar administration) consistently demonstrate neuroprotective effects on retinal ganglion cells (RGCs) and confirm good safety and tolerability in humans. These investigations typically employed elevated intraocular pressure models, showing that NGF preserves RGC integrity despite increased intraocular pressure, or they monitored intraocular pressure as a safety parameter and found no NGF-induced reduction in intraocular pressure [225,226,227,228,229,230,231]. Several preclinical glaucoma models (e.g., induced ocular hypertension in rats) showed that topical or intraocular NGF administration did not cause further intraocular pressure elevation; instead, it promoted retinal ganglion cell survival, inhibited apoptosis, and supported functional recovery [226]. An open-label clinical pilot treated three progressive glaucoma patients with murine NGF eye drops (200 μg/mL, four times daily for seven weeks) and observed improvements in visual acuity, contrast sensitivity, visual field, and electrophysiological measures—with no reported intraocular pressure worsening [227].

Beyond its well-known neurotrophic actions, NGF also plays roles in non-neural wound healing [232]. In skin and oral wound models, it promotes angiogenesis via VEGF-Akt-NO signaling pathways, and NGF participates in angiogenesis and myofibroblast differentiation during tissue repair [228].

Yet, there is no study that has shown the application of NGF alone (e.g., local NGF injection or topical NGF) to directly produce tendon healing in an experimental or clinical model. However, specifically, after tendon injury, NGF expression in tenocytes and surrounding nerve fibers increases, promoting reinnervation and sensory nerve ingrowth via TrkA signaling [233]. NGF (10 µg given via osmotic pump over 7 days) improves ligament healing [234] by promoting reinnervation and angiogenesis and producing scars with enhanced mechanical properties.

### 2.8. Insulin (and the IGF Axis)

Insulin promotes corneal epithelial cell migration, proliferation, and nerve recovery largely via PI3K–Akt signaling [235,236]. A considerable number of studies supported the corneal ulcer healing effect in in vitro and animal work, and human application [235,237,238,239,240,241,242,243]. Regardless of long-term safety and optimal formulations remaining understudied, reported topical use has been well tolerated in published series with minimal systemic effects [244]. This is evidence that topical insulin/IGF signaling can aid corneal nerve regeneration—relevant in neurotrophic keratopathy [245].

Considering the influence on corneal neovascularization, topical insulin promotes epithelial healing but has not been commonly reported to cause corneal neovascularization in human series. Multiple human case series and small trials report faster epithelial closure with topical insulin and few immediate vascular complications, although most studies are small and with short follow-up [242]. Moreover, topical application of insulin encapsulated by chitosan-modified PLGA nanoparticles alleviates alkali burn-induced corneal neovascularization [246].

On the other hand, as a particular point, insulin (native) and soft-tissue wound healing are consistently established, as multiple animal and human studies show topical/local insulin accelerates wound closure and upregulates angiogenic mediators (VEGF, eNOS, SDF-1α) in cutaneous wounds—mechanistically relevant to tendon repair because angiogenesis supports early healing [247]. Moreover, there is a direct effect of insulin on tendon cell effects. At least in vitro, insulin can affect tendon progenitor/tenocyte proliferation and differentiation [248]. Clinical case series and small randomized trials using topical insulin for corneal epithelial healing (typical regimens ~0.5 U/drop QID or diluted solutions) report no change in IOP during follow-up and no systemic glycemic effects in most reports.

Animal wound-healing studies with topical insulin similarly report no intraocular pressure change [249,250]. Systematic reviews and meta-analyses of cohort studies report an increased risk of open-angle glaucoma in people with diabetes (pooled RRs ≈ 1.3–1.5). This is one of the strongest and most reproducible epidemiologic signals [251].

On the other hand, the evidence of insulin and the IGF axis promoting corneal epithelial cell migration, proliferation, and nerve recovery largely via PI3K–Akt signaling [234] should be seen with evidence that IGF-1 alone could not increase corneal ulcer healing and had only an effect combined with substance P (which is also ineffective when given alone) [252,253]. Furthermore, in a rat model of corneal neovascularization, suppression of IRS-1—a downstream mediator of IGF-1 signaling—led to inhibited vessel growth, implying that IGF-1/IRS-1 signaling contributes to corneal angiogenesis [254]. In the cornea, IGF-1 is listed among several angiogenic mediators capable of disrupting corneal avascularity when dysregulated [57].

Considering the influence on intraocular pressure, IGF-1 can be responsible for the increased intraocular pressure (at least in a synergistic mechanism). Namely, daily intraocular pressure of IGF-1 and FGF-2 caused dose-dependent increases in intraocular pressure (chick models), although given alone, they are both ineffective [255].

On the other hand, the beneficial effect of IGF-1 on tendon healing is focused on several reviews (i.e., [256]) postulated in rat and equine studies, using local application [257,258,259,260], and in humans [261]. However, some points remained, both in animal studies (i.e., no gross histologic difference reported; biomechanical failure load not clearly improved [257]) as well as in clinical trials (not improved tendon synthesis, structure, or patient-reported outcomes beyond exercise alone [262]).

### 2.9. Summary of Corneal Ulcer Healing Therapy in Terms of “Triad” Approach (Corneal Ulcer Healing↔Corneal Neovascularization↔Intraocular Pressure)

Summarizing, the majority of available evidence for several corneal wound healing agents remains preclinical. This is particularly true for ascorbate, fibronectin, hyaluronic acid, MMP inhibitors, FGF, and insulin/IGF-1, where supportive data largely derive from animal models, pilot studies, or small uncontrolled clinical series [242,263,264,265]. Only NGF (NGF, cenegermin) [266] and, to a lesser extent, EGF [267] and hyaluronic acid [133] have been tested in larger randomized clinical settings with regulatory approval achieved for NGF (Table 2). Note, Table 2 concisely summarizes the key topical agents trialed to enhance corneal wound healing [133,242,264,265,267,268,269,270], along with their mechanisms, representative PubMed citations (PMID/DOI), and evidence levels.

Importantly, no study to date has evaluated these therapies within an integrated experimental framework that considers the dynamic interplay of corneal ulcer healing, corneal neovascularization, and intraocular pressure [1,2], the corneal ulcer healing/corneal neovascularization/intraocular pressure “triad”, or links ocular outcomes to avascular-tissue (tendon) healing as a validation axis. On the other hand, most preclinical effects of these agents have firmly established the framework of corneal ulcer, including various mechanisms and multiple factors, and different standpoints to approach corneal ulcer healing. Therefore, these open the possibility for novel attempts even outside of the current frame [1,2]. Consequently, there is an additional, yet resolving, and novel approach, and the novel BPC 157 evidence, also preclinical. Thereby, it can be acknowledged that BPC 157 therapy accordingly arrives with healing corneal ulcer, counteraction of corneal neovascularization, and counteraction of the increased intraocular pressure [3,9,35,36,37,38,39,40], linking corneal phenomena to avascular tendon healing [42,43,44,45,46,47,48,49], as a first exemplar framing of corneal ulcer healing, corneal neovascularization, and intraocular pressure as a unified “triad” within a unified cytoprotection framework.

To implement the cytoprotection concept in eye therapy, in particular in corneal ulcer healing, perceiving BPC 157 corneal ulcer healing capability as a whole was carried out, respecting corneal healing agents as a class. Commonly, comparing corneal ulcer healing with the “triad” (corneal healing/corneal neovascularization/intraocular pressure relation) and healing in another avascular tissue like tendon, under a cytoprotection umbrella, these agents, ascorbate, fibronectin, hyaluronic acid, MMP inhibitors, EGF, FGF, NGF, and insulin/IGF-1, all revealed the particular “triad” relations, and a connection or no connection with tendon healing. These differences, now revealed, with the caveat that some direct evidence may be lacking, appeared to be likely specific to any of these agents’ activities organization (Table 3) (e.g., ↑ healing = beneficial, ↓ neovascularization = beneficial, ↑ intraocular pressure = harmful).

There, as an indicative point, using the application of the agents as a proof, there is ascorbate, along with the noted particular BPC 157 relation, where the corneal ulcer ↑ ↔ corneal neovascularization ↓ ↔ intraocular pressure ↓ ↔ linked to other avascular tissues healing (i.e., tendon) ↑-relation occurred as a shared general most favorable effect. These appear to be close to the insulin course (corneal ulcer ↑ ↔ corneal neovascularization not affected (0) or ↓ ↔ intraocular pressure not affected (0), and tendon healing ↑) as well as to NGF (NGF corneal ulcer healing occurs as a sole corneal ulcer ↑, given non-affected (0), the corneal neovascularization and intraocular pressure, and likely ↑ tendon healing (given the noted ligament healing)). Some outcomes from such a course occurred with hyaluronic acid (corneal ulcer ↑ ↔ corneal neovascularization ↓ ↔ intraocular pressure ↑ and tendon healing ↑). Contrarily, different, perilous interrelations occurred, likely specific, for fibronectin (corneal ulcer ↑ ↔ corneal neovascularization ↑↔ intraocular pressure ↑), MMP-inhibitors (corneal ulcer ↑ ↔ corneal neovascularization ↓↔ intraocular pressure ↑ and tendon healing ↓), EGF (corneal ulcer ↑ ↔ corneal neovascularization ↑↔ intraocular pressure not affected (0), and ↑ tendon healing), and FGF (corneal ulcer ↑ ↔ corneal neovascularization ↑ ↔ intraocular pressure ↑, and ↑ tendon healing). IGF-1 is different (corneal ulcer healing not affected (0) (application alone)↔corneal neovascularization ↑ ↔ intraocular pressure ↑, and ↑ tendon healing).

Thus, from the viewpoint of the adequate and safe healing of the corneal ulcer, with “triad” presentation, and connection with tendon healing, as an additional key, the so far acknowledged large range of the framework of corneal ulcer, including various mechanisms and multiple factors, and different standpoints to approach corneal ulcer healing, should be further clarified and specifically pointed out. Whatever the specific mechanism behind this, some points should be favored (i.e., ascorbate) or presumed (hyaluronic acid, insulin, NGF), while others could be possibly discarded (i.e., fibronectin, MMP-inhibitors, EGF, FGF, and IGF-1), as a triad-based approach may better capture therapeutic efficacy and safety in the complex environment of ocular surface disease.

To support these favorable points, BPC 157 therapy for corneal ulcer healing is also combined with maintaining transparency and counteracting corneal lesions after complete corneal abrasion, dry eye after lacrimal gland removal, or cornea insensitivity after topical ophthalmic anesthetics, as well as counteracting the decrease in the tear volume [3,9,35,36,37,38,39,40]. In a broader wound context, i.e., skin wound healing, regularly used to support the beneficial effect of growth factors on corneal ulcer healing, BPC 157 therapy, given alone, without any carrier addition, unlike growth factors, as reviewed, heals both external and internal wounds [22,23,24,25]. These include also in diabetic animals [271,272] a beneficial effect, whatever the route of application. Moreover, as an important analogy for corneal lesions healing, BPC 157 wound healing is a particular one [22,23,24,25]. This can be suggested, providing simultaneous healing of different tissues, as noted with both internal and external fistula healing, and various anastomosis healing, given the specific beneficial outcome, toward the reestablishment of the original presentation (i.e., fistula closure, concomitant damages counteracted, healed intestinal anastomosis, and adhesions counteracted) [24,25]. Note, without therapy, some of the fistulas could be lethal in rats [24]. Likewise, the tendon healing improvement, in vivo and in vitro [42,43,44,45,46,47,48,49,76,77], is well-grounded and reviewed [4,10,11,66,67,273], given the beneficial effect after tendon transection, and detachment from bone or muscle [42,43,46,47].

## 3. Corneal Neovascularization

### 3.1. The Antiangiogenic Agents in Terms of the “Triad” Approach (Corneal Ulcer Healing↔Corneal Neovascularization↔Intraocular Pressure)

The antiangiogenic agents include endostatin and endostatin analogs (neostatin, canstatin, tumstatin), plasminogen activator inhibitor-1 (PAI-1), and serine protease inhibitors, retinal pigment epithelium-derived factor (PEDF), angiostatin, thrombospondin 1,2 (TSP-1, TSP-2, respectively), and interferon (IFN)-α [1,56,57,58,59].

#### 3.1.1. Endostatin

Suppressed corneal neovascularization accompanied by enhanced corneal healing outcomes was consistently reported in numerous studies which have evaluated the application of endostatin (i.e., in its native protein form, engineered or modified variants, or via gene delivery) in corneal ulcer and corneal injury models [274,275,276,277,278].

No single primary paper was identified that directly and explicitly reports the application of endostatin (in a corneal/ulcer model) inducing an increase in intraocular pressure while simultaneously counteracting corneal neovascularization.

Counteraction of corneal neovascularization appeared in ocular gene-therapy/intravitreal/subretinal studies that express endostatin and/or angiostatin (RetinoStat^®^ and related preclinical work) [279,280,281,282]. The preclinical papers report effective suppression of choroidal neovascularization in laser corneal neovascularization models and no persistent, vector-attributable increases in intraocular pressure in the preclinical safety programs (intraocular pressure was explicitly monitored in the Binley safety/biodistribution studies) [280,281,282].

No paper directly tested the application of exogenous endostatin (local injection into tendon or systemic administration) and reported a measured decrease in tendon healing as the primary outcome. The closest direct experimental evidence [283,284,285] supports that excess endostatin can delay skin wound healing [283,284] and that endostatin is produced in tendon and regulated by mechanical load [285]. These findings provide plausible mechanistic support that exogenous endostatin could impair tendon healing via anti-angiogenic and matrix-modifying actions—but they are not direct tendon-intervention studies [283,284,285].

#### 3.1.2. PAI-1

There is no study in which the recombinant PAI-1 (or other exogenous PAI-1 preparations) was applied to the cornea (topically, subconjunctivally, intracorneally, intrastromally, intravitreally, or systemically) and shown to reduce corneal neovascularization. On the other hand, the supporting evidence is obtained in context/dose dependence in ocular compartments other than the cornea. The expression of PAI-1 mRNA was analyzed in human and murine choroidal neovascularization by RT-PCR. The influences of increasing doses of recombinant PAI-1 were evaluated by daily intraperitoneal injections in PAI-1 (-/-) and wild-type animals with a model of laser-induced corneal neovascularization [286]. PAI-1 is pro-angiogenic at physiological concentrations and anti-angiogenic at higher concentrations [287]. PAI-1 facilitates retinal angiogenesis in a model of oxygen-induced retinopathy [288].

The plasminogen/PA system in corneal ulceration and wound healing is largely reviewed [1,289]. However, only one in vivo study, carried out with subconjunctival injection of recombinant Serpine1 (PAI-1) in streptozotocin-diabetic mice, shows that exogenous (PAI-1/Serpine1) applied to the eye accelerated corneal epithelial wound closure [290]. Further supporting studies are in vitro demonstrations that exogenous PAI-1 promotes adhesion and chemotactic migration of human corneal epithelial cells—mechanistic support for the in vivo findings [291]. Likewise, exogenous PAI-1 enhances keratinocyte migration and protects against plasminogen-induced detachment—supportive evidence from epidermal models (not cornea) [292].

There is no study to show that exogenous PAI-1 administration by itself (topical, intracameral, intravitreal, subconjunctival, or systemic) would increase intraocular pressure in animal models or humans. However, considering combining PAI-1 with increased intraocular pressure, PAI-1 contributes to glaucoma pathogenesis; the evidence includes the elevated PAI-1 in eyes with glaucoma/aqueous humor, and PAI-1 expression in anterior-segment tissues that can affect outflow; thus, the plasminogen/plasmin system (and tPA) modulates outflow/intraocular pressure [293,294,295,296].

Although there is no study in which the application of PAI-1 (plasminogen-activator-inhibitor-1) alone (topical, local or systemic) on tendon healing was tested, PAI-1 is associated with fibrotic adhesions after tendon injury, while genetic deletion or therapeutic inhibition of Serpine1 (PAI-1) reduces adhesions and improves remodeling [297,298,299].

#### 3.1.3. PEDF

Exogenous PEDF (full-length protein or PEDF-derived peptides/fragments) promotes corneal epithelial/limbal wound healing and/or inhibits corneal neovascularization [300,301,302,303,304].

Direct experimental evidence that PEDF application increases outflow resistance was provided by Rogers et al., 2013 [305], who showed that purified PEDF decreases outflow facility in enucleated mouse eye perfusions and increases transendothelial electrical resistance of Schlemm’s canal endothelial cells. Thus, PEDF application could raise intraocular pressure (because decreased outflow facility→higher intraocular pressure). Moreover, PEDF is present and is differentially abundant in the aqueous humor in inflammatory and glaucomatous states [306,307,308].

Application of PEDF or PEDF-derived peptides promotes tendon healing/tendon regeneration (or closely related tissue-regenerative effects) [309,310,311]. Illustratively, topical/local delivery of a PEDF-derived short peptide in an alginate hydrogel promoted Achilles tendon regeneration in a rat full-thickness injury model (improved histology, better collagen alignment, and increased tensile strength) [309].

#### 3.1.4. Angiostatin

There is compelling evidence for the application of angiostatin reducing corneal neovascularization, and which reports, explicitly or implicitly, improvements in corneal ulcers/wound healing [312,313,314,315].

There is no report of angiostatin-induced intraocular pressure increase.

Likewise, there is no report of the application of angiostatin on tendon healing. Interestingly, angiostatin-functionalized collagen scaffolds suppress angiogenesis; histologically, after 8 weeks, the scaffolds with angiostatin exhibited fewer inflammatory cells and more collagen matrix formation [316].

#### 3.1.5. TSP-1, TSP-2

Foulsham et al. (2019), in a comprehensive review, summarizes TSP-1′s roles in ocular surface homeostasis, corneal wound healing, infectious keratitis, and anti-(lymph) angiogenesis; also, it notes the benefit of topical recombinant TSP-1 in mouse models [317]. Classic proof-of-concept that exogenous TSP-1 promotes corneal epithelial closure was adding purified TSP-1 to wounded corneal explants that accelerated re-epithelialization, while anti-TSP-1 antibody inhibited it [318]. In a dry-eye epitheliopathy model, topical recombinant TSP-1 reduced dendritic-cell maturation, inflammatory cytokines, and clinical severity [319]. The effects of TSP1 on hypoxia-induced damages and wound-healing activity in human corneal epithelial (HCE) cells demonstrate that TSP-1-induced exosomal proteins attenuate hypoxia-induced paraptosis in corneal epithelial cells and promote wound healing [320].

Thus, in consideration of evidence of TSP-1 or TSP-2 application alone inducing corneal ulcer healing, existing studies suggest that these proteins play significant roles in regulating angiogenesis and extracellular matrix assembly, processes crucial for corneal wound healing [1,57,58].

There is no direct study showing that exogenous application of TSP-1 or TSP-2 alone (e.g., topical, intracameral, intravitreal, or systemic) directly induced an intraocular pressure rise. However, there is genetic and knockout evidence showing TSPs modulate IOP, and mechanistic/cellular work showing exogenous TSP-1 can reduce outflow facility in perfused anterior segments (which would be consistent with intraocular pressure elevation) [321,322,323,324].

Likewise, there is no direct study showing that exogenous application of TSP-1 or TSP-2—i.e., giving TSP-1 or TSP-2 alone (topically, intratendinous, systemic, etc.)—affects tendon-healing outcome in an in vivo tendon model. However, there is other evidence consistent with a worsening effect on tendon healing. TSP isoforms regulate connective-tissue/tendon structure and healing responses (genetic (knockout/transgenic) studies), and elevated endogenous TSP-2 (e.g., in diabetes) is associated with impaired healing, given that TSP-1/TSP-2 can drive profibrotic/anti-angiogenic/ECM-remodeling pathways that could worsen repair [325,326,327,328,329].

#### 3.1.6. IFN-α

Human case reports/series [330,331,332,333,334] report corneal ulcer healing or remission following IFN-α (systemic or topical) in selected cases (Mooren’s ulcer, refractory herpetic keratitis, HCV-associated peripheral ulceration).

Miller et al. (1993) showed regression of experimental iris neovascularization with systemic IFN-α in primates (strong anti-angiogenic signal in that model) [335], while others showed limited efficacy in the treatment of corneal neovascularization, with subcutaneous IFN-α [336] or subconjunctival (IFNα-2a) [337].

There are clinical reports that document increased intraocular pressure/glaucoma occurring during interferon-α (including peg-IFN α) therapy [338,339,340,341].

Primary manuscripts showed that application of IFN-α alone could clearly neither improve nor worsen tendon healing, had no effect on adhesion or repair strength, or described antifibrotic/anti-contraction effects in vitro [342,343,344,345].

### 3.2. Summary of the Antiangiogenic Agents in Terms of the “Triad” Approach (Corneal Ulcer Healing↔Corneal Neovascularization↔Intraocular Pressure)

Furthermore, to implement the cytoprotection concept in eye therapy, in particular in corneal neovascularization counteraction, perceiving BPC 157 corneal ulcer healing capability as a whole was carried out, respecting the antiangiogenic agents, endostatin, PAI-1, PEDF, angiostatin, TSP-1/2, IFN-α, as a class. Commonly, comparing corneal ulcer healing with the “triad” (corneal ulcer healing ↑ ↔ corneal neovascularization ↓ ↔ intraocular pressure ↓ ↔ linked to other avascular tissues healing (i.e., tendon)↑-relation under a cytoprotection umbrella (e.g., ↑ healing = beneficial, ↓ neovascularization = beneficial, ↑ intraocular pressure = harmful), these antiangiogenic agents revealed the particular different corneal healing/corneal neovascularization/intraocular pressure “Triad” relations, connected or no connected with tendon healing, likely specific for any of these agents’ activities organization (Table 4). However, some caveats should be present given the lack of some direct evidence. Endostatin exhibited particular relations (corneal ulcer healing ↑ remained not determined↔corneal neovascularization ↓ ↔ intraocular pressure not affected (0)↔linked to other avascular tissues healing (i.e., tendon) ↓ (based on the effect on skin lesion). PAI-1 exhibited distinctive relations (corneal ulcer healing ↑ ↔ corneal neovascularization ↓↑ (pro-angiogenic at physiological concentrations and anti-angiogenic at higher concentrations)↔intraocular pressure not affected (0) or ↑ (PAI-1 contributes to glaucoma pathogenesis)↔linked to other avascular tissues healing (i.e., tendon) ↓). PEDF also exhibited distinctive relations (corneal ulcer healing ↑ ↔ corneal neovascularization ↓ ↔ intraocular pressure ↑ ↔ linked to other avascular tissues healing (i.e., tendon) ↑). Angiostatin also evidenced a distinct course (corneal ulcer healing ↑ ↔ corneal neovascularization ↓ ↔ intraocular pressure not affected (0)↔linked to other avascular tissues healing (i.e., tendon) not determined (0) or ↑ (given angiostatin-functionalized collagen scaffolds). TSP-1, TSP-2 exhibited a distinct course (corneal ulcer healing ↑ ↔ corneal neovascularization ↓ ↔ intraocular pressure not affected (0) or ↑ (genetic studies)↔linked to other avascular tissues healing (i.e., tendon) not determined (0) or ↓ (TSP-1/TSP-2 can drive profibrotic/anti-angiogenic/ECM-remodeling pathways)). IFN-α also evidenced a distinct course (corneal ulcer ↑ ↔ corneal neovascularization ↓ ↔ intraocular pressure not affected (0)↔linked to other avascular tissues healing (i.e., tendon) not determined (0)).

Thus, it is commonly acknowledged that endostatin, PAI-1, PEDF, angiostatin, TSP-1/2, and IFN-α should act from the viewpoint of corneal neovascularization, and counteraction, in order to obtain adequate and safe healing of the corneal ulcer [56]. However, with “triad” presentation and connection with tendon healing, as an additional key, this could not be conceptualized. It seems that the various mechanisms and multiple factors, and different standpoints to approach corneal ulcer healing and corneal neovascularization, are all not resolving, providing the extent of perilous events commonly present. On the other hand, whatever the specific mechanism involved, by contrasting different antiangiogenic agents (endostatin, PAI-1, PEDF, angiostatin, TSP-1/2, IFN-α), their divergent cross-tissue effects were highlighted. This helps expose the “fragmentation problem” emphasized earlier.

### 3.3. Treatment of Corneal Neovascularization in Terms of the “Triad” Approach (Corneal Ulcer Healing↔Corneal Neovascularization↔Intraocular Pressure)

Treatment of corneal neovascularization is either topical (corticosteroids and non-steroidal anti-inflammatory drugs (NSAIDs), cyclosporine A, anti-VEGF drops) or surgical (laser photocoagulation, diathermy/fine needle cauterization, superficial keratectomy, subconjunctival injections of anti-VEGF drugs, amniotic membrane transplantation, corneal transplantation). However, corneal neovascularization treatment is not always effective and can produce side effects [56].

#### 3.3.1. Corticosteroids

Experimental/clinical data show that topical/systemic corticosteroids—when used alone or before appropriate antimicrobial therapy—can delay or aggravate corneal ulcer/keratitis healing [346,347,348,349,350,351].

Corticosteroids (topical/subconjunctival/systemic) or angiostatic steroid formulations—used alone—inhibit or counteract corneal neovascularization [352,353,354,355,356,357].

Administration of corticosteroids alone (topical, local, systemic, or intraocular) can induce elevation of intraocular pressure (steroid-induced ocular hypertension/steroid glaucoma) [358,359,360,361,362,363,364,365,366].

Administration of corticosteroids alone (local/intratendinous, subacromial, systemic in some models) can impair or aggravate tendon healing, reduce tensile strength, or cause degenerative changes [367,368,369,370,371,372,373,374].

#### 3.3.2. NSAIDs

There is consistent general evidence for the aggravation of corneal ulcer healing due to the application of NSAIDs [375,376,377,378,379,380].

Topical/systemic NSAIDs alone (cyclooxygenase inhibitors) reduced or inhibited corneal neovascularization [381,382,383,384,385].

There is no report that topical/systemic NSAIDs given alone cause clinically meaningful intraocular hypertension. On the other hand, there is strong evidence that NSAIDs antagonize, interfere with, or modify the intraocular pressure-lowering effect of prostaglandin analogues (and in some designs produce small relative increases vs. controls, typically ~1 mmHg) [386,387,388,389,390,391].

Finally, there is evidence that NSAID (COX inhibitor) administration alone aggravated (impaired/delayed or weakened) tendon or tendon-to-bone healing [392,393,394,395,396].

#### 3.3.3. Cyclosporine A

There are many manuscripts (human and animal) reporting that cyclosporine A application—topical or systemic—was associated with corneal ulcer healing [397,398,399,400,401,402].

Likewise, many manuscripts report that cyclosporine A (topical or systemic) application alone counteracted or inhibited corneal neovascularization (animal models and experimental studies) [397,398,399,400,401,402,403,404,405,406,407].

Application of cyclosporine A alone counteracted or reduced increased intraocular pressure (chiefly by substituting cyclosporine A for topical corticosteroids in steroid-induced ocular hypertension/post-keratoplasty glaucoma, and in steroid-intolerant patients) [408,409,410].

There is no report of cyclosporine on tendon healing. However, several mechanistic papers (e.g., [411]) show that cyclosporine A interferes with collagen folding/processing and cultured-cell collagen production; multiple animal wound-healing studies [412,413,414,415] report delayed or impaired healing or altered granulation/collagen deposition. A focused tendon histology study [416] reported no morphologic change in tendons after long-term low-dose cyclosporine A (but did see muscle changes).

#### 3.3.4. Anti-VEGF Drugs

Peer-reviewed experimental manuscripts are showing that anti-VEGF (primarily bevacizumab given subconjunctivally in alkali-burn models) accelerated restoration of the basement membrane, reduced haze, and improved corneal transparency—i.e., promoted corneal wound/ulcer healing [417,418]. Contrarily, several animal studies [419,420,421] and some clinical observations [422] show that topical anti-VEGF (eye-drop formulations, higher concentrations, longer duration) can delay epithelial closure, cause epitheliopathy, or increase stromal remodeling [419,420,421]. Dose, route (subconjunctival vs. topical), timing, model (alkali burn vs. epithelial debridement), and species matter a lot. These healing controversies (promoted vs. impaired) were explained, claiming that anti-VEGF can reduce neovascularization and inflammation (useful), but VEGF also plays roles in normal wound healing, so blocking it may impair epithelial regeneration depending on how it is used [423,424].

In treating corneal neovascularization, studies collectively support the efficacy of topical anti-VEGF therapies, particularly ranibizumab and bevacizumab, in human clinical case series/trials [425,426,427]. There are animal experimental studies demonstrating inhibition [428,429,430,431], and ranibizumab studies [431,432] showing hem- and lymphangiogenesis inhibition.

Evidence of increased (sustained or persistent) intraocular pressure following intravitreal anti-VEGF injections is presented in many reports and case series [433,434,435,436,437,438,439,440].

The evidence that anti-VEGF/tendon healing goes with high VEGF/neoangiogenesis correlates with impaired biomechanical properties [441], and improved tendon-related outcomes after anti-VEGF (VEGF-blocking) intervention (in vivo or clear mechanistic evidence) [442,443,444]. Notably, local injections of an anti-VEGF antibody (B20.4-1-1) given late in the healing course caused a temporary reduction in mechanical properties at an early time point (day 14) when vascularity was reduced—followed by later improvement at a subsequent time point [445].

#### 3.3.5. Summary of Treatment of Corneal Neovascularization in Terms of the “Triad” Approach (Corneal Ulcer Healing↔Corneal Neovascularization↔Intraocular Pressure)

Furthermore, to implement the cytoprotection concept in eye therapy, in particular in corneal neovascularization counteraction by treatment of corneal neovascularization (corticosteroid, NSAIDs, cyclosporine A, anti-VEGF drops), perceiving BPC 157 corneal ulcer healing capability as a whole was carried out, respecting the mentioned agents used in the treatment of corneal neovascularization as a class. Commonly, comparing corneal ulcer healing with the “triad” (corneal ulcer healing ↑ ↔ corneal neovascularization ↓ ↔ intraocular pressure ↓ ↔ linked to other avascular tissues healing (i.e., tendon) ↑-relation under a cytoprotection umbrella (e.g., ↑ healing = beneficial, ↓ neovascularization = beneficial, ↑ intraocular pressure = harmful), corticosteroid, NSAIDs, cyclosporine A, and anti-VEGF agents appear to be outside, and revealed no common effect. Contrarily, there are particular different corneal ulcer healing/corneal neovascularization/intraocular pressure “Triad” relations, connected or not connected with tendon healing, likely specific to any of these agents’ activities organization (Table 5). Corticosteroids, with counteraction of corneal neovascularization, exhibited particular detrimental relations (corneal ulcer healing ↓ ↔ corneal neovascularization ↓ ↔ intraocular pressure ↑ ↔ linked to other avascular tissues healing (i.e., tendon) ↓). NSAIDs exhibited a similar chain of events (corneal ulcer healing ↓ ↔ corneal neovascularization ↓ ↔ intraocular pressure ↑ ↔ linked to other avascular tissues healing (i.e., tendon) ↓). On the other hand, cyclosporine and anti-VEGF drugs could be regarded as “fragmented and context-dependent” rather than strictly negative. Cyclosporine A exhibited a different course (corneal ulcer healing ↑ ↔ corneal neovascularization ↓↔ intraocular pressure ↓ ↔ linked to other avascular tissues healing (i.e., tendon) ↓). Anti-VEGF drops exhibited an additional different course, which is quite complex (corneal ulcer healing ↑ (reduced neovascularization and inflammation) ↓ (inhibition of normal healing (VEGF blocked))↔corneal neovascularization ↓↔ intraocular pressure ↑ ↔ linked to other avascular tissues healing (i.e., tendon ↓ (early points), ↑ (late points)) (Table 5).

Thus, with respect to corneal ulcer healing/corneal neovascularization (↓ neovascularization ≈ ↑ corneal ulcer healing), corticosteroid and NSAIDs act differently, and depart to opposite relation (↓ corneal neovascularization ≠ ↑ corneal ulcer healing). There is a delayed corneal ulcer healing along with counteracted corneal neovascularization. Likewise, the effects of cyclosporine A, and anti-VEGF agents clearly preclude the suggested mechanisms as a way to achieve safe corneal ulcer healing. Furthermore, with a full “triad” presentation (↑ intraocular pressure (corticosteroid, NSAIDs, anti-VEGF agents), ↓ intraocular pressure (cyclosporine)), and connection with tendon healing (quite consistently impaired tendon healing (except for the later interval with anti-VEGF), as an additional key, this could not be conceptualized, and divergent cross-tissue effects were highlighted.

On the other hand, as mentioned before, these favorable points (↓ neovascularization ≈ ↑ corneal ulcer healing) may be more supported by BPC 157 therapy for corneal ulcer healing [3]. Besides being combined with maintaining transparency and counteracting corneal lesions after different injuries [3,38,39,40], but favoring an angiogenesis healing effect in different tissue healing [4], in particular in those avascular, like tendon and ligament [42,43,44,45,46,47,48,49], as emphasized before, there is additional evidence of an orchestrated effect, depending on the tissue, time, and injury [4]. Notably, recently, cornea healing “angiogenic privilege” was reviewed as a part of BPC 157 pleiotropic effects [4]. It appears as a therapy and safety key, targeting angiogenesis and NO’s cytotoxic and damaging actions, but maintaining, promoting, or recovering their essential protective functions, a special beneficial effect controlling and modulating angiogenesis and the NO-system, thus, along with advanced tendon and other tissues healing as a proof of the concept [4]. Consistent with such a modulatory and controlling role, BPC 157 therapy strongly counteracted the adverse effects of both corticosteroids [4,10,43,45] and NSAIDs [4,10,446]. Moreover, consistent with such a role, BPC 157 retrobulbar application directly mitigated the detrimental retrobulbar application of NO-blocker, L-NAME in rats, and L-NAME-induced retinal ischemia [37]. Fundoscopy demonstrated strong generalized irregularity of the diameter of blood vessels with severe atrophy of the optic nerve, and extremely poor presentation of the choroidal blood vessels. Consistently, there was a progressively worsened course, degeneration of ganglion cells, and narrowing of vascular lumina in the nerve cell layer (Factor VIII immunohistochemistry), marked damage in the inner plexiform and inner nuclear layers, accompanied by reduced retinal thickness and evidence of complete retinal injury. BPC 157 therapy may induce a prompt recovery, either given 20 min after L-NAME or 48 h after L-NAME, and markedly improved animal behavior on days and weeks after retrobulbar application of L-NAME [37]. Unlike growth factors [447], consistent with Folkman’s key concept (inhibited corneal neovascularization, inhibited tumor growth) [448,449,450,451], in the human melanoma cell line, BPC 157 inhibits the VEGF effect [73], attributed to controlling the VEGF system, as well [4,49,70,71,72]. Furthermore, in mice with C26 colon adenocarcinoma, BPC 157 counteracted tumor cachexia and severe muscle wasting, corrected deranged muscle proliferation and myogenesis, counteracted weight loss, and markedly prolonged survival [79].

## 4. Treatment of Glaucoma in Terms of the “Triad” Approach (Corneal Ulcer Healing↔Corneal Neovascularization↔Intraocular Pressure)

Recently, it was pointed out that such a wide cytoprotection agenda in glaucoma therapy might also be distinctive from the focused background of the alpha 2-agonists, beta-blockers, inhibitors of carbonic anhydrase, or parasympathomimetics, ROCK/Rho-kinase (Rho) inhibitors, and prostaglandin derivatives [9].

### 4.1. Alpha 2-Agonists

Randomized controlled trial data on corneal ulcer healing due solely to alpha 2-agonists used in glaucoma therapy are not available. On the other hand, many manuscripts, case reports, case series, and an in vitro mechanistic study document adverse corneal effects from topical alpha 2-agonist antiglaucoma agents (i.e., brimonidine and multi-drug regimes including brimonidine)—evidence that these agents can aggravate or complicate corneal disease/healing, and can cause corneal epithelial/stromal inflammation, sterile infiltrates, pseudodendritic lesions, neovascularization, and persistent stromal opacity [452,453,454,455,456,457,458].

For both commonly used topical alpha-2 agonists (brimonidine, apraclonidine), the peer-reviewed clinical literature consistently documents that intraocular pressure lowering begins within about 1 h and peaks at ~2–3 h after a topical dose [459,460,461,462,463,464].

Considering the possible effect on tendon healing, although there is no study of the direct effect on tendon injury of alpha 2-adrenergic agonists used in anti-glaucomatous therapy, alpha 2A-adrenoceptors have been detected in ocular tissues (ciliary epithelium, trabecular meshwork, retina) and in extraocular tissues (e.g., tendons, skin fibroblasts). Stimulation may affect cell proliferation, vascular tone, and inflammation [465,466,467,468,469,470]. Like for corneal pathology, the overall weight of evidence indicates that alpha 2-agonist activity is detrimental (negative effect) for tendon healing. Specifically, direct experimental alpha 2-agonist stimulation in tendon induces tendinosis-like hypercellularity [471]. Human biopsy/immunohistochemistry studies [472,473] suggest adrenergic signaling sustains tendinopathy pathology. Additionally, a systematic review linked sympathetic/adrenergic activity with tendinopathy [474]. Cutaneous wound healing in a knockout mouse model demonstrated that alpha 2 receptor signaling slows repair; absence improves healing [470].

### 4.2. Beta-Blockers

Beta-blockers used for glaucoma (timolol or related beta-adrenergic antagonists) accelerated or improved corneal epithelial wound/ulcer healing [475,476,477].

Likewise, there is direct evidence that beta-blocker agents used in glaucoma therapy (timolol, propranolol, etc.) counteract corneal neovascularization [478,479,480]. Contrarily, far less consistent appear to be the negative or mixed reports where beta-blockers (timolol, propranolol, betaxolol) were tested against corneal neovascularization but did not consistently inhibit or sometimes had no effect, or were less robust than the comparative agent [481,482,483].

Topical beta-blockers (e.g., timolol) begin to lower intraocular pressure within ~15–30 min after instillation; the maximal intraocular pressure reduction is usually reached within ~1–3 h, and the effect commonly persists ~12–24 h with single doses [484,485,486].

Considering the possible effect on tendon healing, there is no study of the direct effect on tendon injury of beta-blockade used in anti-glaucomatous therapy. However, there are beneficial effects of beta-blockade on inflammation, re-epithelialization, angiogenesis, and bone formation—mechanisms that overlap with phases of tendon repair (inflammation modulation, matrix remodeling, vascular responses). The animal and human burn/fracture clinical data [487,488,489,490] are especially load-bearing for systemic beta-blocker effects on repair. The topical timolol case series and small randomized scar studies [491,492,493] show local beta-blockade can promote epithelial/dermal repair when applied to skin wounds.

### 4.3. Carbonic Anhydrase Inhibitors

No rigorously controlled, PubMed-indexed studies have directly demonstrated that topical or systemic carbonic anhydrase inhibitors negatively affect corneal ulcer epithelial re-epithelialization as a primary outcome. However, there is a consistent set of PubMed-indexed clinical reports, case series, and clinical studies showing that topical carbonic anhydrase inhibitors (dorzolamide, brinzolamide ± combinations) can cause or worsen corneal edema/endothelial decompensation or surface epithelial damage in eyes with compromised corneal endothelium or ocular surface. These effects would reasonably aggravate healing of a corneal ulcer in those vulnerable eyes. Multiple case series and case reports document corneal edema/endothelial decompensation temporally associated with the use of topical carbonic anhydrase inhibitors (dorzolamide, brinzolamide), sometimes reversible on cessation and in some reports irreversible—clearly relevant because corneal edema and endothelial failure worsen outcomes and delay epithelial healing of ulcers [494,495,496]. Randomized/controlled and short-term studies show measurable increases in central corneal thickness or changes in corneal hydration control in patients with endothelial compromise or cornea guttata after dorzolamide, i.e., a physiologic basis for impaired corneal deturgescence that would impede ulcer healing [497,498]. Clinical reviews/practice notes advise caution or prefer systemic carbonic anhydrase inhibitors in the setting of infected or compromised corneas because topical carbonic anhydrase inhibitors may be toxic to damaged endothelium or ocular surface—practical guidance consistent with the clinical reports above [499].

No PubMed-indexed controlled trials have proven that carbonic anhydrase inhibitors directly cause corneal neovascularization. However, there are a small number of PubMed-indexed case reports/case series and one narrative/case-report review in which corneal stromal sterile infiltration with neovascularization occurred in patients who were using topical antiglaucoma medications that included a carbonic anhydrase inhibitor (brinzolamide or dorzolamide)—typically in the context of multiple topical agents [500,501,502].

Intraocular pressure reduction with topical carbonic anhydrase inhibitors (dorzolamide, brinzolamide) starts within ~1 h. Intraocular pressure reduction with systemic carbonic anhydrase inhibitors (acetazolamide, methazolamide) starts within 1–2 h (acetazolamide) and 2–4 h (methazolamide) [503].

There are no PubMed-indexed animal experiments or clinical studies in which topical or systemic carbonic anhydrase inhibitors used at glaucoma doses were shown to aggravate or improve tendon healing. However, certain carbonic anhydrase isoforms are upregulated during skin wound healing, and the activity of carbonic anhydrases can influence re-epithelialization—useful background for carbonic anhydrase roles in tissue repair [504]. More specifically, carbonic anhydrases (CAs) are involved in many pathological conditions, and the overexpression of both CA9 and 12 in inflamed joints has been recently reported. Consequently, a selective CA9/12 inhibition could be a feasible strategy for improving tendon recovery after injury, evidenced in an in vitro study on human tenocytes showing carbonic anhydrase-targeting compounds can change inflammatory signaling in tendon cells [505].

### 4.4. Muscarinic Agents (Including Pilocarpine)

In controlled animal studies, muscarinic agents (including pilocarpine) used in anti-glaucomatous therapy improve corneal epithelial defect/ulcer healing [506,507]. Contrarily, there is a lack of effect of 2% pilocarpine on corneal epithelial healing in rabbits [508]. Moreover, an in vitro study on primary human corneal stromal cells found dose- and time-dependent cytotoxicity of pilocarpine (apoptosis, membrane permeability, DNA fragmentation, caspase activation) at concentrations above ~0.625 g/L [509]. Likewise, using rabbit corneal endothelium, in vitro perfusion, and electron microscopy, there is a dose-related endothelial toxicity (cell shrinkage, nuclear margination, cytoplasmic vacuolation). This is the basis for statements that pilocarpine (especially at higher concentrations/exposure) can harm corneal endothelium in animal models [510]. These results are consistent with other studies [511,512]. Additionally, there is a reported pilocarpine-associated allograft rejection in post-keratoplasty patients [513]. On the other hand, measuring corneal epithelial healing and acetylcholine content in the corneal epithelium found that higher acetylcholine levels were associated with faster healing, thus, a positive correlation between endogenous acetylcholine level and wound healing [514].

Consequently, as a result, no studies directly demonstrate that muscarinic agents used in glaucoma treatment either inhibit or promote corneal neovascularization. Still, the mentioned Massry & Assil (1995) study in a clinical case series (three cases) described a temporal association between initiation of topical pilocarpine and corneal graft rejection—inflammatory events that can be accompanied by neovascularization in some contexts [513].

Considering the lowering of the increased intraocular pressure, pilocarpine acts within minutes to an hour and lasting several hours (formulation dependent). Short-acting acetylcholinesterase inhibitors (physostigmine) show rapid daytime effects (onset in tens of minutes and peak within 1–2 h). Echothiophate produces miosis within an hour but exhibits a delayed peak intraocular pressure decrease (~24 h) and very prolonged duration (days–weeks). Carbachol intracameral application acts essentially immediately (minutes) in the intraoperative setting with topical preparations lowering intraocular pressure over the first hours [514,515,516,517,518].

No direct manuscripts show that muscarinic agents (pilocarpine, carbachol, or other) used in glaucoma therapy either counteracted or improved tendon healing. However, although without testing pilocarpine or other glaucoma-used muscarinic drugs as tendon therapeutics, some papers provide related mechanistic/preclinical evidence (acetylcholine/acetylcholinesterase-inhibitor). They show cholinergic stimulation (exogenous acetylcholine or increasing acetylcholine by inhibiting acetylcholinesterase), and that muscarinic receptor activation in tenocytes can enhance tenocyte proliferation or improve bone–tendon (enthesis) healing in animal models [519,520]. Therefore, muscarinic signaling may facilitate tenocyte proliferation through a cellular mechanism.

### 4.5. Rho-Kinase Inhibitors

Rho-kinase inhibitors are (or are the same class as) drugs used in glaucoma therapy (ripasudil, netarsudil), which can improve corneal wound/ulcer or epithelial/endothelial healing [521,522,523,524].

The evidence about Rho-kinase inhibitors and corneal neovascularization suggests the reduction in corneal neovascularization. Of the ROCK inhibitors that are actually used clinically for glaucoma, ripasudil (K-115) has clear, peer-reviewed evidence showing a reduction in corneal neovascularization in an experimental corneal transplant model (see Inomata et al.) [525]. Several other ROCK inhibitors (notably fasudil and the research compound Y-27632) have preclinical evidence for anti-angiogenic effects (in vitro or in animal CNV/retinal models)—these support the mechanism but are not (in most countries) glaucoma drugs in routine clinical use [526]. For netarsudil (an FDA-approved glaucoma drug), the evidence includes corneal hemorrhage or corneal vascularization temporally associated with netarsudil use—so the clinical/real-world corneal effects of netarsudil are mixed and may even include adverse vascular events in some settings [527].

Illustrative is a Phase 2 clinical trial (0.4% ripasudil in primary open angle glaucoma/ocular hypertension therapy patients), where peak intraocular pressure lowering was observed 2 h after instillation, while a trough (lowest effect before the next dose) was recorded just before the next instillation [528].

There are no manuscripts showing that the Rho/ROCK inhibitors that are used clinically for glaucoma (i.e., topical ripasudil or netarsudil) improve tendon healing. The glaucoma-drug literature is focused on ocular tissues (trabecular meshwork, cornea, Tenon’s fibroblasts, etc.), not musculoskeletal tendon repair. Mechanistic, preclinical evidence exists: multiple lab studies show that inhibiting ROCK (commonly with Y-27632 or related inhibitors) affects tendon stem/progenitor cell mechanics and can enhance tenogenic differentiation in vitro. Those findings suggest a mechanistic plausibility that ROCK modulation could help tendon repair, but they do not prove that the clinical glaucoma drugs (ripasudil/netarsudil) will do so in vivo [529,530].

### 4.6. Latanoprost

Latanoprost, in terms of corneal ulcer healing, exhibited a dual effect. It improved corneal ulcer healing [531,532,533,534,535,536] consistent with commonly acknowledged evidence that just preservative-free or alternative-preservative prostaglandin formulations (or cationic-emulsion latanoprost) have reduced corneal toxicity and better wound-healing profiles. Contrarily, latanoprost (especially benzalkonium chloride (BAK)-preserved formulations) can aggravate corneal surface injury or delay corneal wound healing/recur herpetic keratitis [531,535,537,538,539,540].

Latanoprost, in terms of corneal neovascularization, exhibited a dual effect. Prostaglandin analogues can promote corneal angiogenesis [539,540,541,542,543,544]. Illustrative as direct evidence that prostaglandin analogues can promote corneal angiogenesis in certain models is the study in a rat corneal micropocket model [545], where prostaglandin formulations produced the most prominent angiogenic stimulatory effect among several antiglaucoma drugs tested. Consistently, as mentioned, in a case report of stromal corneal neovascularization temporally associated with latanoprost therapy, corneal neovascularization regressed after discontinuation—which underscores that in real patients latanoprost/its formulation can be linked to corneal neovascularization [541]. On the other hand, preservative-free/cationic-emulsion latanoprost formulations have markedly better ocular-surface tolerance and promote corneal epithelial healing and reduced surface inflammation compared with BAK-preserved latanoprost, which may reduce the risk of secondary corneal neovascularization as an indirect effect [531,532,533,536].

Measurable intraocular pressure reduction begins within a few hours after topical dosing. Clinical single-dose studies report the average time to 50% of maximal intraocular pressure reduction (a practical “onset” measure) of about 6.0 h [546].

No direct evidence currently links topical latanoprost to affect tendon healing. However, a dual role could be suggested. This was based on evidence that local PGE_2_ application increased some structural strength metrics, illustrating that prostaglandin signaling modulates tendon remodeling and mechanics [547]. On the other hand, an in vitro study demonstrated that low levels of PGE_2_ can support tendon remodeling and healing, while high levels of PGE_2_ impair tendon stem cell proliferation and push them toward non-tenocyte lineages (bone, fat, cartilage), which is detrimental to tendon repair [548].

### 4.7. Summary of Treatment of Glaucoma in Terms of the “Triad” Approach (Corneal Ulcer Healing↔Corneal Neovascularization↔Intraocular Pressure)

Furthermore, to implement the cytoprotection concept in eye therapy, in particular in the treatment of glaucoma (alpha 2-agonists, beta-blockers, carbonic anhydrase inhibitors, muscarinic agonists (including pilocarpine), Rho-kinase inhibitors, latanoprost), perceiving BPC 157 corneal ulcer healing capability as a whole was carried out, respecting the mentioned agents used in the treatment of glaucoma as a class. Commonly, comparing corneal ulcer healing with the “triad” (corneal ulcer healing ↑ ↔ corneal neovascularization ↓ ↔ intraocular pressure ↓ ↔ linked to other avascular tissues healing (i.e., tendon) ↑-relation under a cytoprotection umbrella (e.g., ↑ healing = beneficial, ↓ neovascularization = beneficial, ↑ intraocular pressure = harmful), most of agents appear to be outside, while beta-blockers and probably Rho-kinase inhibitors appear to be inside, and revealed no common effect. This can be seen with the reduction of aqueous production (alpha 2-agonists, beta-blockers, carbonic anhydrase inhibitors) as well as with increases of uveoscleral outflow of aqueous humor (muscarinic agonists (including pilocarpine), Rho-kinase inhibitors, latanoprost). Thus, with the caveat that some direct evidence is lacking, there are particular different corneal ulcer healing/corneal neovascularization/intraocular pressure “Triad” relations, connected or not connected with tendon healing, likely specific for any of these agents’ activities organization (Table 6). Alpha 2- agonist, with counteraction of increased intraocular pressure, exhibited particular detrimental relations (corneal ulcer healing ↓ ↔ corneal neovascularization ↑ ↔ intraocular pressure ↓ ↔ linked to other avascular tissues healing (i.e., tendon) ↓). Carbonic anhydrase inhibitors exhibited a distinct chain of events (corneal ulcer healing ↓ ↔ corneal neovascularization ↑ ↔ intraocular pressure ↓ ↔ linked to other avascular tissues healing (i.e., tendon) ↑ (effect supposed as carbonic anhydrases are involved in many pathological conditions). On the other hand, β-blockers’ counteraction of increased intraocular pressure exhibited a promising course (corneal ulcer healing ↑ ↔ corneal neovascularization ↓ ↔ intraocular pressure ↓ ↔ linked to other avascular tissues healing (i.e., tendon) ↑ (based on the effect on skin lesion)). Muscarinic agents (including pilocarpine) provide a counteraction of increased intraocular pressure along with a particular course (i.e., dual effect on corneal ulcer healing) (corneal ulcer healing ↑ ↓ ↔ corneal neovascularization ↑ ↔ intraocular pressure ↓ ↔ linked to other avascular tissues healing (i.e., tendon) ↑ (enhanced tenocyte proliferation). Rho-kinase inhibitors exhibited a counteraction of increased intraocular pressure along with a course similar to that of beta-blockers (corneal ulcer healing ↑ ↔ corneal neovascularization (corneal ulcer healing ↑ ↔ corneal neovascularization, ripasudil, fasudil ↓, netarsudil ↑ ↔ intraocular pressure ↓ ↔ linked to other avascular tissues healing (i.e., tendon) ↑ (tendon stem/progenitor cell mechanics). Latanaprost-induced lowering of intraocular pressure is frequently accompanied by other dual effects, and thereby, there is a course that cannot be easily estimated (corneal ulcer healing, preservative-free latanoprost ↑, BAK-preserved products ↓ ↔ corneal neovascularization preservative-free latanoprost ↓, BAK-preserved products ↑ ↔ intraocular pressure ↓ ↔ linked to other avascular tissues healing (i.e., tendon, low level of PGE_2_ ↑, high level of PGE_2_ ↓).

Notably, for current glaucoma therapies, although all current glaucoma therapies are acknowledged as potent and effective intraocular pressure-lowering agents, they require substantial time to start and exert their lowering effects and do not share a unifying therapeutic concept. Moreover, combined with the other avascular tissue healing (i.e., tendon), the standard agents used in glaucoma therapy lack the common concept even more, given the corneal ulcer healing/corneal neovascularization/intraocular pressure “triad”. Furthermore, almost any one of the particular concepts, presenting very distinctive drug classes, shows particular discrepancies and limited effectiveness (Table 6). Thereby, for the success of glaucoma therapy, as intraocular pressure-lowering agents, the additional combining of these principles can be indicative since very early times (i.e., pilocarpine and epinephrine [549,550,551], timolol concomitant to epinephrine to enhance the ocular hypotensive effects in many patients [552]). With respect to corneal ulcer healing/corneal neovascularization/intraocular pressure, the “triad”, combined with the other avascular tissue healing (i.e., tendon), the indicated encouraging effect of timolol could be confounded with beta-blockers started with propranolol. Pitfalls encountered were intravenous applications lowering intraocular pressure, avoiding corneal anesthetic properties, which have produced negative effects on tear production, profound dry eye syndrome, subconjunctival fibrosis, and tachyphylaxis [553]. Finally, there is the ambiguous corneal ulcer healing/corneal neovascularization/intraocular pressure, the “triad”, combined with the other avascular tissue healing (i.e., tendon) noted with latanoprost, a resolving prostaglandin analogue. Therefore, the proposed selective activation of the FP receptor in ciliary muscle and adjacent tissues, remodeling of the extracellular matrix, and upregulating matrix metalloproteinases to increase uveoscleral outflow of aqueous humor [41], does not correlate with the proposed cytoprotection context once related to prostaglandins’ cytoprotective role [13,14,15,16,17,18,19,20,21], while sustained reduction in intraocular pressure requires more hours.

Thus, there is a new and more complex point with BPC 157 therapy in glaucomatous rats, and eye therapy, in general [3,9,35,36,37,38,39,40,42,43,44,45,46,47,48,49]. Presenting quite an immediate lowering effect on the increased intraocular pressure, and with respect to the known therapy, achievement, and pitfalls [549,550,551], the proposed BPC 157 cytoprotective therapy can be an additional resolving principle [4,5,6,7,8,9,10,11,12,23,24,25,26]. Notably, it covers the recovery of glaucomatous rats [9,35] and normalized intraocular pressure [35], maintenance of retinal integrity [35,37], recovery of pupil function [36,37], recovery of retinal ischemia [37], counteracting of corneal neovascularization [35], and corneal injuries [3,38,39,40]. In particular, ascertained transparency maintenance [3,38,39,40], counteracting corneal drying, counteracting the loss of corneal sensation, counteracting the decrease in blink rate, and maintaining tear production [39,40] have equal effectiveness for topical and systemic application. The specificity of the therapy [4] of BPC 157 that should be requested is confirmed also in several tendon injury models [42,43,44,45,46,47,48,49].

Thereby, the proposed BPC 157 cytoprotective principle should be more viable in glaucomatous rats. Three episcleral vein cauterizations, and intraocular pressure just exceeding 30 mmHg, rapidly counteracted BPC 157 therapy, which underlines the cytoprotective ability of the pentadecapeptide BPC 157 to further maintain and upgrade endothelium integrity and functioning (for review see [4]), particularly focused on minor vessels during noxious procedures, rapidly upgrading function to substitute the failed major blood vessels. Illustratively, this likely recovered glaucomatous rats, where one episcleral vein remained, and took over the failed function of all the episcleral veins, amid rapid lowering of increased intraocular pressure and subsequent retinal integrity preservation, as proof [9,35]. These findings show, along with a systemic beneficial effect, the recovery of the severe occlusion/occlusion-like syndrome [554,555,556,557,558,559,560,561,562,563,564,565,566,567,568,569,570,571]. There, the prompt activation of collateral pathways, “bypassing vascular key”, i.e., activated azygos vein to direct blood flow delivery, results in the recovery of multiorgan failure syndrome. This occurred in the rats with vascular failure induced by major vessels’ occlusion, peripherally [554,555,556,557,558,559] and centrally [560,561], and other similar noxious procedures [562,563,564,565,566,567,568,569,570,571] that largely affect endothelium function, and multicausal pathology was fully recovered [554,555,556,557,558,559,560,561,562,563,564,565,566,567,568,569,570,571]. There were general blood pressure disturbances (i.e., intracranial (superior sagittal sinus), portal and caval hypertension, and aortal hypotension). The lesions occurred in the brain (including intracerebral and intraventricular hemorrhage), heart (severe arrhythmias, congestion, and endocardial infarction), and lungs (hemorrhage), with congestion in the liver, kidney, and gastrointestinal lesions. Major vessels were congested (i.e., inferior caval vein, superior mesenteric vein), the azygos vein collapsed, venous and arterial thrombosis progressed, peripherally and centrally, and the advanced Virchow triad was fully substantiated. As emphasized, also in the context of the effects noted in BPC 157 eye therapy, these were all attenuated/eliminated by BPC 157 therapy [554,555,556,557,558,559,560,561,562,563,564,565,566,567,568,569,570,571]. Illustratively, major vessel congestion was reversed to normal vessel presentation, the recovered azygos vein reactivated the pathway for direct blood flow delivery, and the vascular failure (and Virchow triad circumstances) was effectively cured [554,555,556,557,558,559,560,561,562,563,564,565,566,567,568,569,570,571]. Furthermore, Fourier transform infrared spectroscopy revealed a rapid change in the lipid content and protein secondary structure conformation within the vessel wall, occurring within minutes, following BPC 157 therapy [572]. This shows support for the vessel’s function even in the worst circumstances. Thus, the BPC 157 beneficial effect of the corneal healing/corneal neovascularization/intraocular pressure “triad” combined with avascular tissue (i.e., tendon) healing occurred as part of its general cytoprotective effect [4].

Finally, we should conclude with resolving transparency, as a particular part of the corneal ulcer healing, and corneal ulcer healing as a part of BPC 157 wound healing therapy [3,4,22,23,24,25]. Notably, inducing simultaneous healing of different tissues specifically depends on the tissue involved, and this should be, in general, along with the cornea healing as a prime point [3,4,22,23,24,25], as a relatively unique tissue in the body, with transparency and a lack of blood vessels [573]. Thus, possessing a somewhat unique defense system [1,2], there is fibrin deposition, a “translucent fibrin clot” in the cornea as an initial scaffold for healing [573]. There, in analogy with specifically demonstrated counteraction of adhesion formation, both prevention and reversal [22,23,24,25], BPC 157 therapy would improve the beneficial course by optimizing clot resolution along with corneal ulcer healing and further improve corneal transparency [3]. Basically, the peritoneum is also unique in its morphology [574] (hemostasis is not relevant [574], as in cornea ulcer healing as well [573]); with damage to the peritoneum, fibrin is deposited. Consequent to fibrin deposition, adhesion formation increases as a sign of necrotic tissue, inflammation, and initial clotting not rapidly removed in a regular course [574,575,576,577,578]. Or, they could be promptly reversed (i.e., by BPC 157 therapy [24,25,575]), as a highlight of an advanced healing course, given that the fibrin matrix is lysed, and normal peritoneal fibrinolytic activity is reestablished [574,575,576,577,578]. Thus, the peritoneum–cornea analogy may serve as an analogous remodeling of fibrin-based repair systems [573,574,575,576,577,578]. Consequently, there is indicative evidence that BPC 157 strongly counteracted adhesion formation following peritoneal excision, and both prevented and reversed those already formed. Likewise, BPC 157 therapy counteracted adhesions along with improved anastomosis healing, and fistula closing, as an effect related to an interaction with the NO-system [24,25,575]. Together, this confirms how BPC 157 may particularly preserve corneal transparency while promoting ulcer healing [3]. In addition, BPC 157 attenuates prolonged bleeding and thrombocytopenia after amputation and anticoagulant or aspirin use as an interaction with the NO-system [83,579], while considering arterial and venous thrombosis counteraction [580], it counteracts the whole Virchow triad as shown in the recovery of the huge pathology in all occlusion/occlusion-like syndromes [554,555,556,557,558,559,560,561,562,563,564,565,566,567,568,569,570,571], mentioned before. Interestingly, when given with aspirin, clopidogrel, or cilostazol, BPC 157 largely rescues thrombocyte function in rats and does not affect coagulation factors [581]. This may be important given that in the cornea, the fibrinolytic system not only participates in the regulation and execution of ECM turnover but also contributes to the control of other physiological functions of the various corneal cell types [573].

## 5. Summary of Corneal Ulcer Therapy, Angiostatic Factors, Neovascularization Therapy, and Glaucoma Therapy in Terms of the “Triad” Approach (Corneal Ulcer Healing↔Corneal Neovascularization↔Intraocular Pressure)

Quite extensive beneficial effects of BPC 157 therapy, as a cytoprotective agent, in eye therapy [3,9,35,36,37,38,39,40], and also in other avascular tissue, tendon healing [42,43,44,45,46,47,48,49], quite extensive even in the cytoprotection terms, apparently interconnected since obtained with the same dose range [3,9,35,36,37,38,39,40,42,43,44,45,46,47,48,49], accord corneal ulcer healing/corneal neovascularization/intraocular pressure relations, as commonly known mutual interrelation [1,2,29,30,31,32,33,34]. Combined tendon healing provides an avascular tissue benchmark that clarifies an agent’s capacity to restore homeostasis in corneal ulcer disease [4]. This enables more mechanism-driven translation, implemented as a “triad”, where cytoprotection serves as the unifying principle that conceptually links corneal ulcer healing, corneal neovascularization, and intraocular pressure regulation, underscoring that therapeutic effects are not isolated but interconnected. Preclinical studies with BPC 157 therapy, as a cytoprotection agent, illustrate this integration (i.e., the corneal ulcer healing (e.g., ↑)↔corneal neovascularization (e.g., ↓)↔intraocular pressure (e.g., ↓) relation is integrated in one agent’s effect). The extent of integration that could be achieved was investigated by mapping standard therapeutic agents used for corneal ulcers, neovascularization, or glaucoma onto this “triad” and linking them with tendon healing.

This new approach challenges the standard therapies, which are typically designed to address single targets, with the standard agents’ background intended to act in “fragments”. This raises the possibility that these fragmented approaches could be reversed conceptually toward the corneal ulcer healing/corneal neovascularization/intraocular pressure “triad”, and other avascular tissue healing, within an integrated cytoprotection framework. As emphasized before, mapping was conducted despite the caveats due to the absence of some direct evidence, heterogeneity of the models and endpoints, and the majority of mapping being preclinical. Included were the ascorbate, fibronectin, hyaluronic acid, metalloproteinase inhibitors, EGF, FGF, NGF, insulin, and IGF-1 (corneal ulcer healing, Section 2.9 (Table 3)), the antiangiogenic agents (endostatin, PAI-1, PEDF, angiostatin, TSP-1, TSP-2, IFN-α (Section 3.2 (Table 4)), corticosteroids, NSAIDs, cyclosporine A, anti-VEGF drops (treatment of corneal neovascularization (Section 3.3.5 (Table 5)), and alpha-agonists, beta-blockers, carboanhydrase inhibitors, muscarinic agonists, Rho-kinase inhibitors, and prostaglandin analogues (glaucoma, Section 4.7, Table 6). To better illustrate the cytoprotection triad, all of the agents were grouped according to their shared phenotypic profiles (Table 7). Consequently, particular relations between the agents appeared, proving that within corneal ulcer healing/corneal neovascularization/intraocular pressure “triad” relations combined with tendon healing as a prime benchmark of orchestrated avascular tissue healing (i.e., resolved cornea’s “angiogenic privilege”), BPC 157, ascorbate, and insulin appear to uniquely fulfill the entire triad and tendon healing without trade-offs (Table 7). Latanoprost, due to its consistent dual effects (except for consistently lowering intraocular pressure), could not be accommodated. Notably, however, for the cytoprotection background, BPC 157 strongly counteracted the adverse effects of insulin overdose application [582]. This suggests a broad cytoprotective buffering potential, given that BPC 157 is very safe (in toxicology (including ocular-specific safety) studies LD1 not achieved [4,583], no adverse effects in clinical trials [62,63,64,65,584]).

Furthermore, it remains to see the significance of the evidence that beta-blockers and Rho-kinase inhibitors closely follow (although with narrower evidence bases) as well as the significance of NGF as primarily reparative, neutral for corneal neovascularization and intraocular pressure. Together, these may be separate pathways to realize the integrative cytoprotection effect. Latanoprost remains a special issue. The other agents seem to be more (providing some adverse outcome as a shared point) or less (providing some beneficial effects combined, i.e., corneal ulcer healing/tendon healing, despite other adverse outcomes) outside the cytoprotection “umbrella”. Hyaluronic acid and PEDF support tendon/corneal healing but may raise intraocular pressure. Cyclosporine A and MMP inhibitors support corneal healing but impair tendon repair. Fibronectin, EGF, FGF, and IGF-1 promote corneal healing and tendon but simultaneously drive corneal neovascularization and intraocular pressure elevation. Classical anti-angiogenics (endostatin, PAI-1, TSP-1/2, IFN-α) suppress corneal neovascularization but impair repair. Possibly, overlapping effects, shared phenotypic patterns, given that corneal healing/corneal neovascularization/intraocular pressure, combined with tendon healing, should be specific for each of the agents, may suggest shared negative points toward the negative outcomes (i.e., increased intraocular pressure, corneal neovascularization, failed corneal ulcer healing, etc.). On the other hand, summarizing the shared phenotypic patterns can immediately provide an insight into which agents fully, partially, or poorly align with cytoprotection. Finally, to avoid a complex discussion about a multitude of factors involved, for instance, in corneal ulcer pathology, the agents’ effects speak for themselves, even without speaking. Thus, the agents’ profiles, when summarized in this triad framework, clearly reveal their alignment—or misalignment—with cytoprotection, and consequently, their therapy potential.

## 6. Conclusions

In conclusion, to better resolve corneal ulcer healing, we introduced a novel “triad” (corneal ulcer healing↔corneal neovascularization↔intraocular pressure), as a conceptual framework in ocular therapy, and extended it to avascular tissues such as tendon and cytoprotection; as the shared conceptual anchor, therapeutic effects are not isolated but interconnected. Within this framework, corneal ulcer healing↔corneal neovascularization↔intraocular pressure “triad” properly interrelated, BPC 157 therapy, as a cytoprotection agent therapy, has shown in preclinical studies positively interconnected beneficial effects. There is the ability to rapidly normalize intraocular pressure in glaucomatous rats, preserve retinal integrity, restore pupil function, promote transparent corneal healing, and counteract both corneal neovascularization and dry eye. Likewise, cornea healing “angiogenic privilege” along with advanced tendon healing as proof of the healing concept, as consistent effects, are documented in tendon injury models, underscoring its cytoprotective specificity. This novel approach was substantiated by mapping standard therapeutic agents specifically used for corneal ulcer healing, or neovascularization therapy, or glaucoma onto this triad and linking them to tendon healing. The mapping of different therapeutic domains under one unifying triad reveals for each class a shared framework of different “triad” presentations, likely specific to the agent’s effect, connection, or no connection with other avascular tissue healing. This would unify disparate therapeutic observations but also highlight gaps and inconsistencies across existing drug classes. These findings encourage further translational research into the cytoprotection concept, cytoprotection as the unifying principle, and BPC 157, in particular, as the first exemplar, potential clinical application.

## Figures and Tables

**Table 1:** Preclinical evidence of BPC 157 in eye therapy.

Condition/Model	Therapeutic Effect of BPC 157	Reference
Glaucoma (rats)	Rapidly lowers elevated intraocular pressure to normal levels; preserves retinal structure	[9,35]
Pupil function disturbances (atropine-induced mydriasis, L-NAME/L-arginine-induced miosis) (rats, guinea pigs)	Restores normal pupil function; effect dependent on NO system interaction	[36]
Retinal ischemia (L-NAME retrobulbar application, rats)	Recovers retinal function and integrity; prevents ischemic damage	[37]
Corneal abrasion (complete epithelial defect, rats)	Promotes rapid healing; maintains corneal transparency	[38]
Corneal ulceration/perforating corneal injury (rats)	Facilitates healing of perforated corneal injury; prevents ulcer progression; counteracts corneal neovascularization	[3]
Dry eye (lacrimal gland removal, rats)	Counteracts tear deficiency and associated damage	[39]
Corneal insensitivity (local anesthetic-induced, rats)	Shortens duration of tetracaine- and oxybuprocaine-induced corneal anesthesia	[40]

**Table 2:** Concise summary of the key topical agents trialed to enhance corneal wound healing, along with their mechanisms, representative PubMed citations (PMID/DOI), and evidence levels. Agents trialed for corneal wound healing: mechanisms, evidence, and key references.

Agent	Mechanism/Therapeutic Role	Representative Reference	Evidence Level
Ascorbic acid (vitamin C)	Antioxidant; promotes collagen synthesis and stromal repair after chemical injuries	[269]	Preclinical (animal) + small clinical series
Fibronectin (autologous)	ECM adhesive protein; supports epithelial migration and adhesion	[264]	Small uncontrolled clinical series
Hyaluronic acid	Hydration; enhances epithelial migration and wound closure	[133]	Controlled clinical trials
MMP inhibitors (e.g., doxycycline)	Inhibit stromal matrix degradation; reduce corneal melting and perforation risk	[265]	Mechanistic + retrospective clinical use
EGF	Stimulates epithelial proliferation and migration	[268]	Randomized clinical trial (historical)
FGF/bFGF	Promotes epithelial and stromal repair; used in post-refractive surgery	[270]	Small clinical studies
NGF (cenegermin)	Promotes nerve regeneration and corneal healing; approved for neurotrophic ulcers	[267]	Pivotal randomized controlled trial (RCT); clinical approval
Insulin/IGF-1 (topical)	Stimulates epithelial proliferation; potential in diabetic/persistent epithelial defects	[242]	Emerging RCTs and pilot clinical data

**Table 3:** Effects of healing agents on corneal ulcer healing, corneal neovascularization, intraocular pressure, and tendon healing (as representative avascular tissue).

Agent	Corneal Ulcer Healing	Corneal Neovascularization	Intraocular Pressure	Tendon Healing (or Other Avascular Tissue)	Notes
BPC 157	↑	↓	↓	↑	Most favorable profile, cytoprotection-consistent
Ascorbate	↑	↓	↓	↑	Similar to BPC 157
Hyaluronic acid	↑	↓	↑	↑	Favorable, cytoprotection-consistent
Insulin	↑	0/↓	0	↑	Close to BPC 157 pattern
Fibronectin	↑	↑	↑	? (no direct evidence)	Limited data on tendon healing
MMP inhibitors	↑	↓	↑	↓	Mixed, less favorable
EGF	↑	↑	0	↑	Divergent ocular vs. tendon effects
FGF	↑	↑	↑	↑	Increased neovascularization/IOP
NGF	↑	0	0	↑ (ligament evidence)	Partial, indirect tendon support
IGF-1	(Unclear/weak ↑)	↑	↑	↑	Problematic, lacks clear corneal evidence

**Table 4:** Antiangiogenic agents in the context of the cytoprotection “triad”.

Agent	Corneal Ulcer Healing	Corneal Neovascularization	Intraocular Pressure (IOP)	Other Avascular Tissues (e.g., Tendon, Skin)
Endostatin	Effect on ulcer healing not determined	↓ (anti-angiogenic)	0 (no clear effect)	↓ (delays in skin/tendon repair reported)
PAI-1	↑	↑ ↓ (pro-angiogenic at physiological levels; anti-angiogenic at higher levels)	0 or ↑ (implicated in glaucoma pathogenesis)	↓ (impairs tendon/skin healing)
PEDF	↑	↓ (anti-angiogenic)	↑ (raises IOP in some models)	↑ (supports tendon/skin healing)
Angiostatin	↑	↓ (anti-angiogenic)	0 (no clear effect)	0/↑ (angiostatin-functionalized scaffolds may enhance tendon repair)
TSP-1/2	↑	↓ (anti-angiogenic)	0 or ↑ (genetic studies suggest possible IOP elevation)	0/↓ (profibrotic, ECM remodeling, anti-angiogenic; may impair tendon healing)
IFN-α	↑	↓ (anti-angiogenic)	0 (no clear effect)	0 (not determined)

**Table 5:** Effects of standard anti-neovascularization therapies mapped to the cytoprotection triad.

Agent	Corneal Ulcer Healing	Corneal Neovascularization (CNV)	Intraocular Pressure (IOP)	Other Avascular Tissues (e.g., Tendon Healing)	Notes
Corticosteroids	↓ (impairs healing)	↓ (suppresses CNV)	↑ (raises IOP)	↓ (delays tendon healing)	Effective for CNV but adverse for ulcer healing, IOP, tendon
NSAIDs	↓ (impairs healing)	↓ (suppresses CNV)	↑ (raises IOP)	↓ (delays tendon healing)	Similar detrimental chain to corticosteroids
Cyclosporine A	↑ (promotes healing)	↓ (suppresses CNV)	↓ (reduces IOP)	↓ (impairs tendon healing)	More favorable for eye, but tendon effects remain negative
Anti-VEGF drops	↑ (via reduced CNV/ inflammation) ↓ (via impaired VEGF-dependent repair)	↓ (suppresses CNV)	↑ (raises IOP)	↓ (early inhibition) ↑ (later recovery)	Dual and context-dependent: beneficial for CNV control but may compromise repair

**Table 6:** Effects of standard glaucoma therapies mapped to the cytoprotection triad.

Drug Class/Agent	Corneal Ulcer Healing	Corneal Neovascularization	Intraocular Pressure (IOP) Effect	Linked Avascular Tissue Healing (e.g., Tendon)	Notes/ Mechanistic Insight
Alpha 2-agonists	↓	↑	↓	↓	Counteracts IOP but may impair corneal and tendon healing; suggests detrimental cytoprotective profile.
Beta-blockers	↑	↓	↓	↑	Reduces IOP and supports healing; aligns well with cytoprotection concept.
Carbonic Anhydrase (CA) Inhibitors	↓	↑	↓	↑	Lowers IOP; corneal healing impaired but tendon healing supported, possibly via broader CA-related pathways.
Muscarinic Agonists (e.g., Pilocarpine)	↑/↓	↑	↓	↑	Dual effect on corneal healing; supports tendon proliferation; complex cytoprotective profile.
Rho-kinase Inhibitors (e.g., Ripasudil, Fasudil, Netarsudil)	↑	↓/↑ (agent-dependent)	↓	↑	Reduces IOP, improves corneal healing, supports tendon stem/progenitor mechanics; generally cytoprotective.
Prostaglandin Analogues (Latanoprost)	↑/↓ (preservative-dependent)	↓/↑ (preservative-dependent)	↓	↑ (low PGE_2_), ↓ (high PGE_2_)	Effects variable depending on preservative; dual effects make cytoprotection assessment complex.

**Table 7:** Grouped agents according to their shared profile within the cytoprotection “triad.” Abbreviations: CNV, corneal neovascularization; IOP, intraocular pressure.

Shared Profile	Agents	Corneal Ulcer Healing	CNV	IOP	Tendon/Skin Healing	Cytoprotection Alignment
Optimal profile (full triad alignment)	BPC 157, Ascorbate, Insulin	↑	↓	↓	↑	Strong; consistent with cytoprotection
Healing +, CNV –, IOP –, Tendon +	β-blockers, Rho-kinase inhibitors	↑	↓	↓	↑	Favorable; close to optimal
Healing +, CNV 0, IOP 0, Tendon +	NGF	↑	0	0	↑	Partial; focused on repair side
Healing +, CNV –, IOP ↑, Tendon +	Hyaluronic acid, PEDF	↑	↓	↑	↑	Mixed; tendon support but IOP concern (PEDF IOP elevation model-dependent)
Healing +, CNV –, IOP 0/↑, Tendon –	Cyclosporine A, MMP inhibitors	↑	↓	0/↑	↓	Partial; corneal support but tendon adverse
Healing +, CNV +, IOP ↑, Tendon +/?	EGF, FGF, IGF-1	↑	↑	↑	↑	Pro-angiogenic bias; not cytoprotection-consistent
Healing +, CNV +, IOP ↑, Tendon ↑	Fibronectin	↑	↑	↑	↑ (no direct evidence)	Pro-angiogenic; tendon repair unclear
Healing +, CNV –, IOP 0, Tendon 0/–	Angiostatin, Thrombospondin-1/2, IFN-α	↑	↓	0	0/↓	Anti-angiogenic but repair-impaired
Healing –, CNV –, IOP ↑, Tendon –	Corticosteroids, NSAIDs, Anti-VEGF	↓	↓	↑	↓	Anti-CNV but globally adverse
Healing 0, CNV –, IOP 0, Tendon–	Endostatin, PAI-1 (high levels)	0	↓	0	↓	Anti-CNV, impairs repair

## Data Availability

No new data were created or analyzed in this study. Data sharing is not applicable.
